# The STIM1-Orai1 pathway of store-operated Ca^2+^ entry controls the checkpoint in cell cycle G1/S transition

**DOI:** 10.1038/srep22142

**Published:** 2016-02-26

**Authors:** Yun-Wen Chen, Yih-Fung Chen, Ying-Ting Chen, Wen-Tai Chiu, Meng-Ru Shen

**Affiliations:** 1 Department of Pharmacology, College of Medicine, National Cheng Kung University, Tainan, Taiwan; 2 Department of Biomedical Engineering, National Cheng Kung University, Tainan, Taiwan; 3 Department of Obstetrics and Gynecology, College of Medicine, National Cheng Kung University, Tainan, Taiwan; 4 Advanced Optoelectronic Technology Center, College of Engineering, National Cheng Kung University, Tainan, Taiwan; 5 Graduate Institute of Natural Products, College of Pharmacy, Kaohsiung Medical University, Kaohsiung, Taiwan; 6 PhD Program in Toxicology, College of Pharmacy, Kaohsiung Medical University, Kaohsiung, Taiwan

## Abstract

Ca^2+^ signaling is important to trigger the cell cycle progression, while it remains elusive in the regulatory mechanisms. Here we show that store-operated Ca^2+^ entry (SOCE), mediated by the interaction between STIM1 (an endoplasmic reticulum Ca^2+^ sensor) and Orai1 (a cell membrane pore structure), controls the specific checkpoint of cell cycle. The fluctuating SOCE activity during cell cycle progression is universal in different cell types, in which SOCE is upregulated in G1/S transition and downregulated from S to G2/M transition. Pharmacological or siRNA inhibition of STIM1-Orai1 pathway of SOCE inhibits the phosphorylation of CDK2 and upregulates the expression of cyclin E, resulting in autophagy accompanied with cell cycle arrest in G1/S transition. The subsequently transient expression of STIM1 cDNA in STIM1^−/−^ MEF rescues the phosphorylation and nuclear translocation of CDK2, suggesting that STIM1-mediated SOCE activation directly regulates CDK2 activity. Opposite to the important role of SOCE in controlling G1/S transition, the downregulated SOCE is a passive phenomenon from S to G2/M transition. This study uncovers SOCE-mediated Ca^2+^ microdomain that is the molecular basis for the Ca^2+^ sensitivity controlling G1/S transition.

Regulation of the cell cycle involves the important processes for cell survival, including the detection and repair of genetic damage as well as the prevention of uncontrolled cell division. The sequence of events that constitute the cell cycle is mainly regulated by extracellular signals and coordinated by internal checkpoints^1^. Two key classes of regulatory molecules, cyclins and cyclin-dependent kinases (CDKs), determine the progress through the cell cycle. In response to various signals, cyclins and CDKs interact to form a complex that activates or inactivates target proteins to orchestrate coordinated entry into the next phase of the cell cycle. For example, cyclin D-CDK4 mainly controls the G1 phase; cyclin E-CDK2 is required to initiate S-phase, while cyclin A-CDK1 and cyclin B-CDK1 control the mitotic phase^2^. The significance of Ca^2+^ signaling for the regulation of cell cycle progression has been highlighted in several types of cells. Intracellular Ca^2+^ transients happen at the wakening from quiescence, at the G1/S transition, during S-phase, and at the exit from mitosis^3^. However, the molecular basis for this Ca^2+^ sensitivity is not known.

Modulation of cytosolic Ca^2+^ levels provides versatile and dynamic signaling that mediates fundamental cellular functions, such as proliferation, migration, gene regulation, and apoptosis^4^. Store-operated Ca^2+^ entry (SOCE) is a major Ca^2+^ entry pathway in non-excitable cells, which involves several steps for activation, including (i) stimulation of G proteins or protein tyrosine kinases activates phospholipase C, which hydrolyzes phosphatidylinositol bisphosphate to release the second messenger inositol-1, 4, 5-trisphosphate (IP3); (ii) binding of IP3 to its receptor in the endoplasmic reticulum (ER) membrane causes rapid and transient Ca^2+^ release from ER lumen; (iii) decreasing ER luminal Ca^2+^ activates SOCE in the plasma membrane^5^,^6^. Two families of proteins, STIM (stromal-interaction molecule) and Orai, are the molecular identities responsible for SOCE activation^7^,^8^. STIM proteins function as an ER Ca^2+^ sensor detecting ER store depletion. Once ER Ca^2+^ is depleted, STIM proteins aggregate into multiple puncta that translocate to the close proximity of plasma membranes. Orai, an essential pore-forming component of SOCE, translocates to the same STIM-containing structures during ER Ca^2+^ depletion and opens to mediate Ca^2+^ entry. STIM proteins are required for the development and function of regulatory T cells and STIM1-deficiency causes several autoimmune diseases and myopathy in human subjects and mouse models^9^,^10^. We and others have demonstrated the important role of STIM1-mediated Ca^2+^ dysregulation involved in tumor development and progression^11^,^12^,^13^. To inhibit STIM1-dependent Ca^2+^ signaling by specifically targeting STIM1 activation and translocation in cancer cells is thus a potential target for cancer therapy^14^.

SOCE has emerged as an important player in cell proliferation, yet the way in which it controls distinct checkpoints in the cell cycle remains elusive. Inactivation of SOCE by STIM1-silencing in smooth muscle cells, cervical and breast cancer cells significantly inhibited cell proliferation by slowing down the cell cycle progression^11^,^13^. During mitosis, phosphorylation of STIM1 leads to ER exclusion from the mitotic spindle, which underlies the suppression of SOCE^15^. Here we show that the activation of SOCE fluctuates during the cell cycle progression, in which the SOCE activity controls G1/S transition but is not necessary for S to G2/M transition.

## Results

### SOCE is necessary for G1/S transition

**Figure 1:**
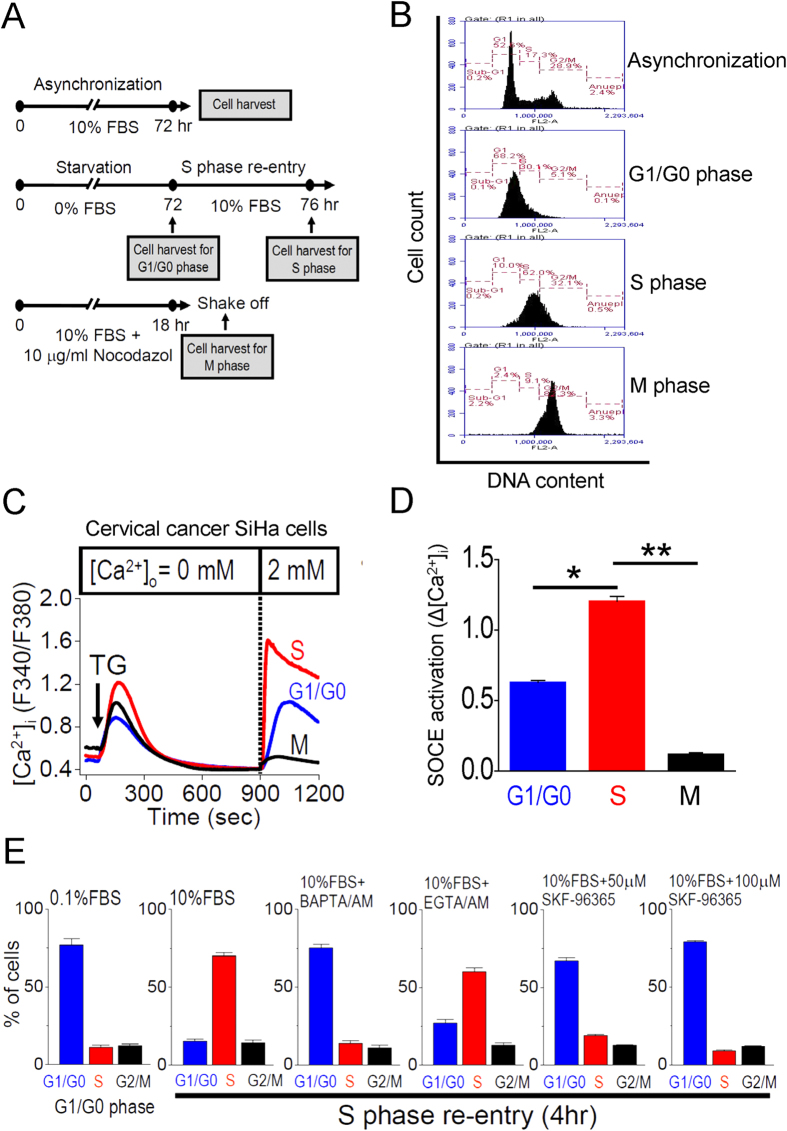
SOCE is necessary for G1/S transition. (**A**) The protocol for cell cycle synchronization. For asynchronization: Cells grew in 10% FBS for 72 hours, then harvested for Fluorescence-Activated Cell Sorting (FACS) measurement to determine cell cycle stages. For G1/G0 phase: Cells grew in 0% FBS for 72 hours, then harvested for FACS measurement. For S phase: Cells grew in 0% FBS for 72 hours and then in 10% FBS for 4 hours before FACS measurement. For M phase, cells grew in 10% FBS with 10μg/ml nocodazole for 18 hours and then were shaken off for collection. (**B**) Representative results of FACS measurements to determine cell cycle stages of cervical cancer SiHa cells under different culture conditions. (**C**) Representative intracellular Ca^2+^ ([Ca^2+^]_i_) measurement in SiHa cells. Each trace is the mean [Ca^2+^]_i_ measurement of at least 100 cells. The SOCE amplitude indicates the rise of [Ca^2+^]_i_ in the replenishment of [Ca^2+^]_o_ from 0 to 2 mM. Arrow, adding 2 μM thapsigargin (TG). (**D**) Quantitative analysis of SOCE at different cell cycle stages. Each value represents mean ± SEM of at least 100 cells. *P < 0.01 **P < 0.001 by unpaired *t* test. (**E**) Quantitative analysis of cell cycle stages, determined by FACS measurement. SiHa cells were incubated with different [Ca^2+^]_i_ chelators (20 μM BAPTA/AM or 100 ng/mL EGTA/AM), or a SOCE inhibitor SKF-96365 (50 or 100 μM) at the beginning of S phase re-entry. Data are the mean ± SEM of three independent experiments.

We first performed a protocol of cell cycle synchronization to determine whether Ca^2+^ signaling plays an important role in cell cycle progression (Fig. 1A). The criteria to determine S, G1/G0 or M phase cells were based on cellular DNA content, detected by flow cytometry (Figs 1B and S1A). We elucidated the functional significance of SOCE activation observed upon cell cycle synchronization by employing single cell [Ca^2+^]_i_ measurement. SOCE activation is triggered by thapsigargin, a sarco/endoplasmic reticulum Ca^2+^ ATPase (SERCA) pump inhibitor. The magnitude of Ca^2+^ influx induced by re-introduction of Ca^2+^ following store depletion (store-operated Ca^2+^ entry: SOCE) was upregulated from G1 to S cell cycle transition and downregulated from S to M phase transition in cervical cancer SiHa cells (Fig. 1C,D) and osteosarcoma U2OS cells (Fig. S1B). The up-regulation of SOCE from G1 to S cell cycle transition was observed using different protocols to activate SOCE (Figs S1D and S2A), and in multiple cell lines of the epithelial or mesenchymal origin (Figs S1C, S2B, S2C). To better understand if the activation of SOCE fluctuates in a cell cycle-dependent manner, we performed another protocol for the cell cycle synchronization (Fig. S3C), in which aphidicolin, a reversible inhibitor of eukaryotic nuclear DNA replication, was used to arrest cells at late G1 phase. The results from two different protocols showed no significant difference in SOCE activation (Fig. S3A–S3E). These results suggest that the upregulated SOCE is likely ubiquitous in cell cycle G1/S transition.

We studied whether Ca^2+^ signaling is necessary for G1/S transition by a specific protocol for cell cycle synchronization (Fig. 1E). BAPTA/AM, the fast [Ca^2+^]_i_ chelator, completely inhibited cell cycle G1/S transition, whereas EGTA/AM, the slow [Ca^2+^]_i_ chelator, showed the moderate inhibition on G1/S transition. SKF-96365, a SOCE inhibitor (Fig. S2D), concentration-dependently arrested cell cycle G1/S transition to a similar inhibitory effect of BAPTA/AM (Fig. 1E). These data indicate that Ca^2+^ microdomain resulting from SOCE is likely the molecular basis for the Ca^2+^ sensitivity controlling G1/S transition.

### SOCE-mediated Ca^2+^ entry is not important for S to G2/M transition

Previous studies have demonstrated the inactivation of SOCE during M phase of the cell cycle in several cell lines, resulting from uncoupling of ER and plasma membrane channels, Orai1 internalization or STIM1 phosphorylation^15^,^16^,^17^. The activation of SOCE was suppressed in M phase, which was demonstrated in cervical cancer SiHa cells (Fig. 1C,D) and osteosarcoma U2OS cells (Fig. S1B). We further showed that the activation of SOCE was progressively decreased when cells moved from S to G2/M phase (Fig. S4). Here we designed a protocol for cell cycle synchronization to study whether the down-regulation of STIM1-mediated Ca^2+^ entry is important for S to G2/M transition (Fig. S5A). Ionomycin is an ionophore used to raise the intracellular level of Ca^2+^. Our results showed that employing ionomycin to maintain Ca^2+^ entry exhibited no significant effect on the cell cycle distribution (Fig. S5B). Furthermore, in the S to G2/M transition, no increased aneuploid was noted in ionomycin-treated cells (Fig. S5B). These results indicate that the SOCE inactivation is not a necessary signaling that controls S to G2/M transition.

### STIM and Orai1 underlying SOCE control G1/S transition

**Figure 2:**
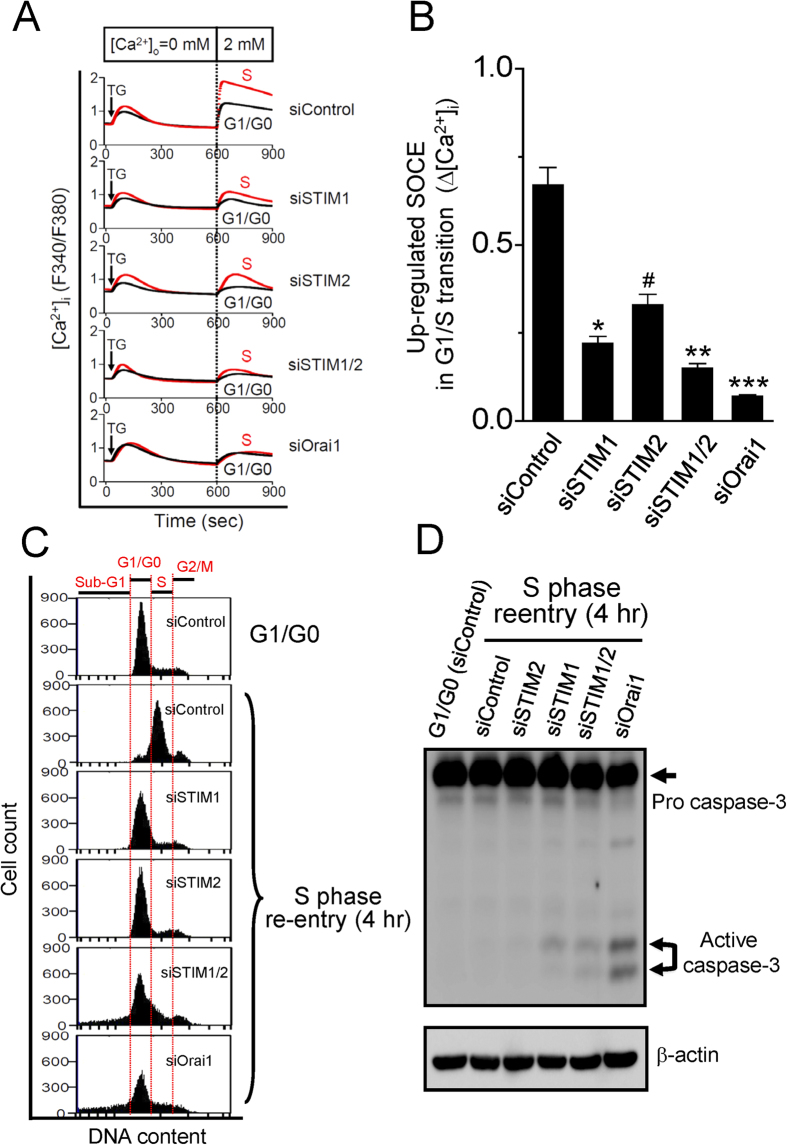
STIM and Orai1 underlying SOCE control G1/S transition. (**A**) Representative [Ca^2+^]_i_ measurement in various conditions. Mean traces of [Ca^2+^]_i_ measurement from at least 30 cells in each condition. Arrow, adding 2 μM thapsigargin. (**B**) Quantitative analysis of up-regulated SOCE in G1/S transition. Data represent mean ± SEM of at least 90 cells from three different experiments. ^#^P < 0.05, *P < 0.01, **P < 0.001, ***P < 0.0001 compared with siControl. (**C**) Representative FACS measurement to determine cell cycle distribution. (**D**) Apoptosis was analyzed by cleaved active caspase-3 expression at different experimental conditions.

As the Ca^2+^ sensor in the ER, STIM1 or STIM2 is capable of triggering a signal cascade leading to SOCE activation. Since SOCE is an immediate downstream target of STIM activation, the important role of STIM family on SOCE activation was studied by knockdown approaches (Fig. S6). STIM1 or STIM2 knockdown by different siRNA duplexes in cervical cancer SiHa cells was accompanied by a significant decrease of SOCE activation (Fig. S7). Double knockdown of STIM1 and STIM2 abolished SOCE activation to a similar inhibitory effect by SOCE inhibitor SKF-96365 (Fig. S7B, S7C). This indicates that both STIM1 and STIM2 contribute to SOCE activation. We further studied whether STIM1 and STIM2 are involved in the regulation of SOCE during G1/S transition. As shown in Fig. 2A,B, up-regulated SOCE in G1/S transition was partially inhibited by specific siRNA for STIM1 or STIM2, respectively. By contrast, double knockdown of STIM1 and STIM2 almost abolished up-regulated SOCE activation in G1/S transition (Fig. 2A,B). Similarly, Orai1-specific siRNA almost completely inhibited the up-regulated SOCE activation in G1/S transition (Fig. 2A,B). We further examined the effect of STIM or Orai1 knockdown on the cell cycle progression (Fig. 2C). Four hours after cell cycle re-entry, 76 + 1.7% of cells in the siControl group were in S phase and less 1% of cells were in sub G1 phase (Fig. 2C). By contrast, 42 + 1.6%, 39 + 1.8%, 46 + 2.1 and 54 + 0.8% of cells were in S phase for siSTIM1, siSTIM2, siSTIM1/2 and siOrai1 groups, respectively (Fig. 2C). Interestingly, the cells in the subG1 phase were significantly increased from 5%, <1% to 5%, 10% and 18% for siSTIM1, siSTIM2, siSTIM1/2 and siOrai1 groups, respectively (Fig. 2C). Moreover, the presence of active caspase-3, one of the major indicative of apoptotic cell death, was also noted in these cells with specific depletion of STIM1, STIM1/2 or Orai1 (Fig. 2D). These data suggest that STIM and Orai1 underlying SOCE control G1/S transition.

### Accelerated and sustained STIM1-trafficking in S phase

**Figure 3:**
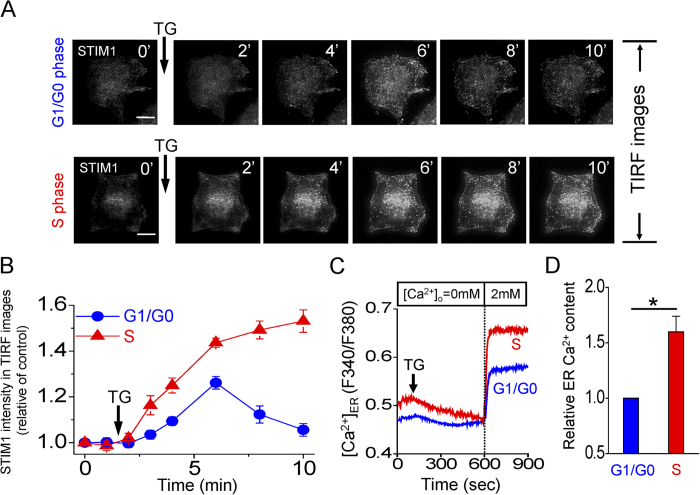
Accelerated and sustained STIM1-trafficking at S phase. (**A**) STIM1 trafficking real-time recorded by total internal reflection fluorescence (TIRF) microscope. TIRF images were from cervical cancer SiHa cells expressed EGFP-STIM1 (Supplementary Movie 1 and 2). Thapsigargin (2 μM) induces the trafficking of STIM1 puncta towards cell periphery. Scale bar, 10 μm. (**B**) Quantitative analyses of the trafficking rate of STIM1 puncta in TIRF images. Each value represents mean ± SEM from at least 15 cells. (**C**) Representative measurements of endoplasmic reticulum Ca^2+^ level ([Ca^2+^]_ER_) in SiHa cells. Mean traces of [Ca^2+^]_ER_ measurement from at least 30 different cells in each experiment. (**D**) Quantitative analyses of [Ca^2+^]_ER_ in G1/G0 and S phase, expressed as the relative of the control group (G1/G0 phase). Each value represents mean ± SEM of at least 60 cells. *P < 0.01.

Upon ER Ca^2+^ depletion, STIM1 was recruited to the plasma membrane to activate SOCE. Although STIM2 is also proposed to be involved in SOCE activation, the mechanism underlying this association remains elusive^18^. Time-lapse fluorescent images of cervical cancer SiHa cells overexpressing fluorescently-tagged STIM1 or STIM2 were employed to study the dynamics of STIM proteins in SOCE activation. As shown in Fig. S8, STIM1 became aggregated and translocated towards cell periphery upon thapsigargin-induced ER Ca^2+^ depletion. In contrast, thapsigargin stimulation did not significantly change the STIM2 puncta and its trafficking toward the juxta-plasma membrane (Fig. S8). These results imply that STIM1 is a major sensor of ER Ca^2+^ levels during SOCE, whereas STIM2 likely plays a housekeeping role. To further study the role of STIM1-mediated SOCE during cell cycle progression in a more quantitative way, we monitored EGFP-STIM1 trafficking by the total internal reflection fluorescence (TIRF) microscopy, which real-time visualizes the fluorescence restricted to within approximately 100 nm from the plasma membrane. For cells at G1/G0 phase, thapsigargin stimulated the aggregation and trafficking of STIM1 toward the proximity of plasma membrane, which reached a plateau at about 5 minutes and then gradually declined (Fig. 3A,B and Supplementary Movie 1). By contrast, thapsigargin induced an accelerated and sustained trafficking of STIM1 in cells at S phase (Fig. 3A,B and Supplementary Movie 2), which can partly explain the upregulation of SOCE at S phase. As a consequence of up-regulated SOCE, ER was refilled with more Ca^2+^, demonstrated by the direct measurement of ER Ca^2+^ using Mag-Fura-2 (Fig. 3C,D).

### Inhibition of SOCE activation alters cyclin E/CDK2 activity

**Figure 4:**
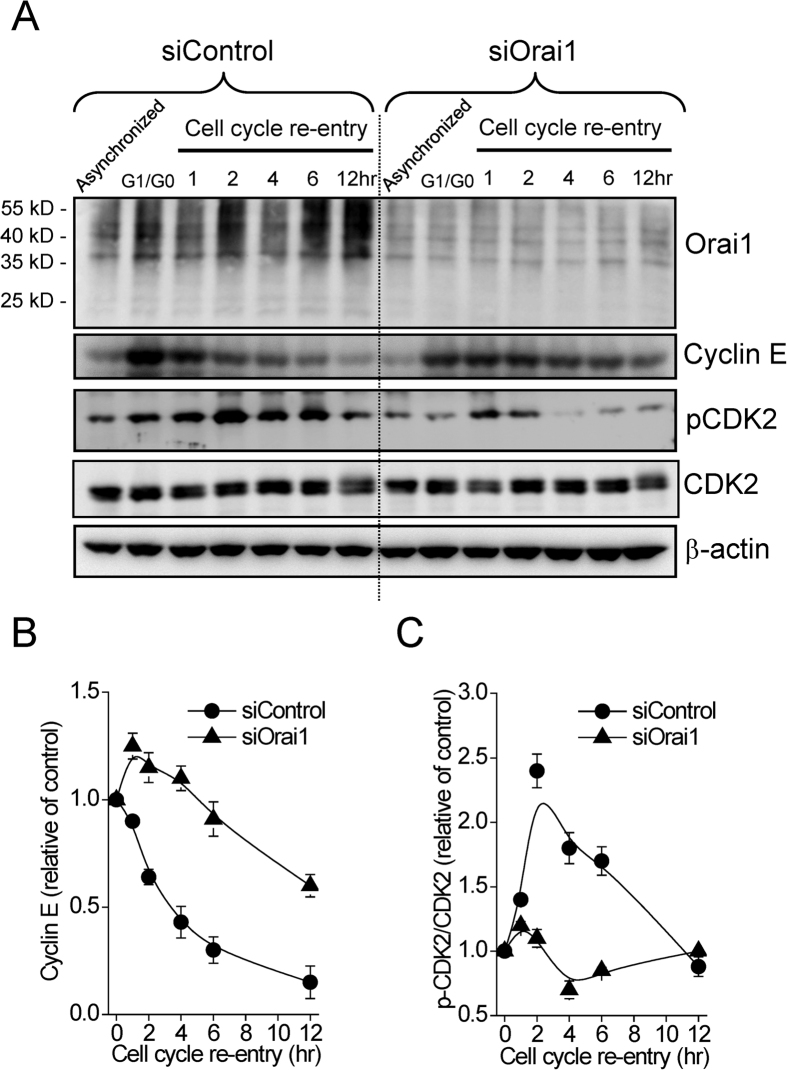
Blockade of SOCE activation alters cyclin E/CDK2 activity. (**A**) The representative immunoblots showing cell cycle-associated proteins, including cyclin E, CDK2 and phosphorylated CDK2 (p-CDK2). Cell lysates were collected from cervical cancer SiHa cells at indicated time points of cell cycle re-entry. (**B,C**) Densitometric quantitative analyses of cyclin E, p-CDK2 and CDK2 level. Expression levels of cyclin E, CDK2 and pCDK2 were normalized against β-actin and compared with the control group (G1/G0 phase). Each point represents mean ± SEM of three different experiments.

As Ca^2+^ entry and cell cycle progression appeared to be associated, we clarified the mechanism by which SOCE-mediated Ca^2+^ entry affects the cell cycle progression. We analyzed the expression of the main cell cycle regulatory proteins and evaluated the activities of specific CDKs that control the G1/S transition. Compared to that of G1/G0 phase, the expression level of cyclin E decreased with time when cells re-entered cell cycle (Figs 4A,B and S9). In contrast, knockdown of Orai1 led to increased expression of cyclin E up to 2 hours of S phase re-entry (Figs 4A,B and S9). During cell cycle progression, cyclin E binds the G1 phase CDK2 that is required for G1/S transition, while CDK2 binding with Cyclin A is required to continue through late S phase^2^. CDK2 activity is regulated through the phosphorylation by CDK-activating kinase (CAK) at Thr 160. To directly test whether SOCE activation is necessary for the Ca^2+^ sensitivity of CDK2 activity, the phosphorylated level of CDK2 (the active form of CDK2) was analyzed. The expression of phosphorylated CDK2 was increased steadily up to 6 hours of cell cycle re-entry (Figs 4A,B and S9). On the other hand, knockdown of Orai1 significantly inhibited the phosphorylation of CDK2 during cell cycle re-entry (Figs 4A,B and S9). Double knockdown of STIM1 and STIM2 also led to the accumulation of Cyclin E and decrement of CKD2 phosphorylation in cervical cancer SiHa cells (Fig. S10). Our previous studies demonstrated an increased p21 protein levels in STIM1-silencing cells, compared with control cells^11^. Here we found that the direct cyclin E/CDK2 substrates such as p27 and phospho-RB were also affected by siOrai1 (Fig. S11).

### STIM1-dependent signaling is important for controlling G1/S transition

**Figure 5:**
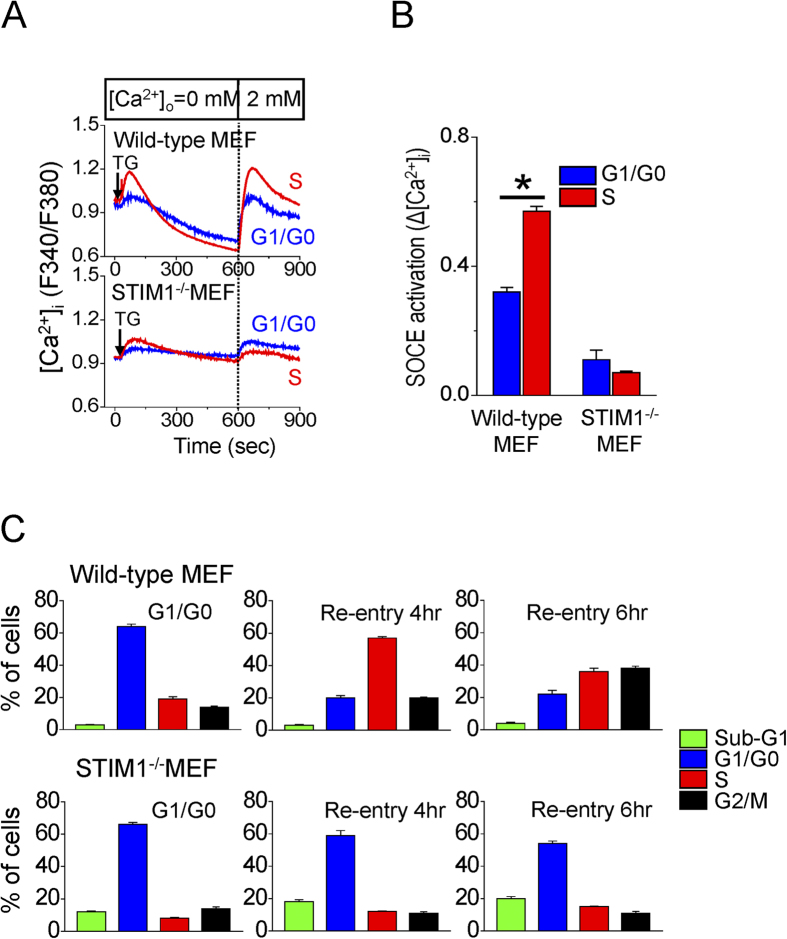
STIM1-mediated SOCE controls G1/S transition. (**A**) Representative [Ca^2+^]_i_ measurement from at least 60 different cells of wild-type and MEF, respectively. The SOCE in wild-type MEF was upregulated from G1 to S cell cycle transition. On the other hand, the activity of SOCE was decreased in STIM1^−/−^ MEF, and there was nearly no upregulation of SOCE activation in G1/S transition. Arrow, adding 2 μM thapsigargin (TG). (**B**) Quantitative analyses of SOCE activation. Each value represents mean + SEM of at least 100 cells. *P < 0.01. (**C**) The cell cycle distribution profile was determined by FACS measurements at G1/G0, 4, and 6 hours of cell cycle re-entry in wild-type MEF and STIM1^−/−^ MEF. Each value represents mean ± SEM (n = 5).

**Figure 6:**
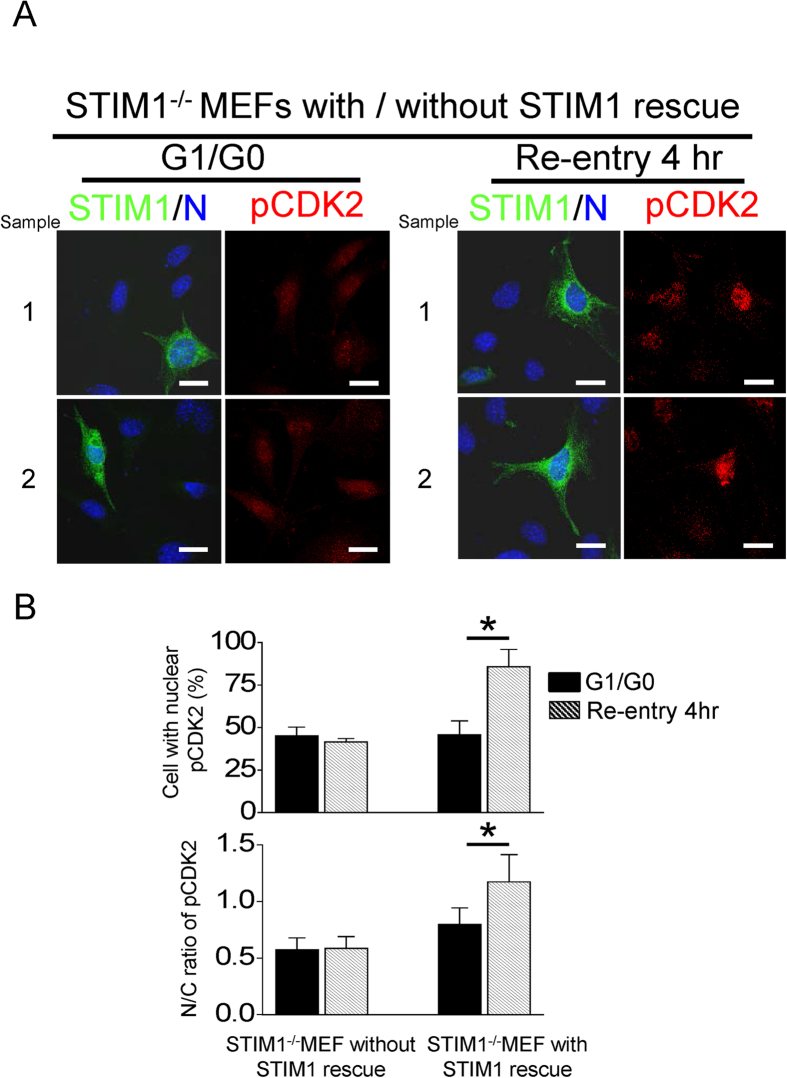
G1/S cell cycle transition depends on the STIM1-Orai1 pathway of SOCE. (**A**) Representative confocal images showing the expression of phosphorylated CDK2 (pCDK2) and STIM1. MEFs lacking STIM1 were re-transfected with EGFP-STIM1 plasmids before cell cycle synchronization. Images were analyzed at G1/G0 and 4 hours of cell cycle re-entry. Nuclei, Hoechst 33258 (blue), pCDK2 (red), STIM1 (green). (**B**) Quantitative analyses of pCDK2 fluorescent intensity at nuclear and cytosolic regions in MEF lacking STIM1 with or without STIM1 rescue. Each value represents mean + SEM from at least 30 different cells. *P < 0.01.

We further utilized mouse embryonic fibroblasts (MEF) lacking STIM1 (STIM1^−/−^) to study the important role of STIM1-mediated SOCE in controlling cell cycle G1/S transition. As shown in Fig. S12A and S12B, the levels of STIM2, Orai1 and phospho-CDK2 were decreased in STIM1^−/−^ MEF cells. SOCE was upregulated from G1 to S cell cycle transition in wild-type MEF (Fig. 5A,B). On the other hand, the activity of SOCE was decreased in STIM1^−/−^ MEF and there was nearly no upregulation of SOCE activation in G1/S transition (Fig. 5A,B). More importantly, the experiments of cell cycle synchronization demonstrated that STIM1 is critical to regulate G1/S transition (Fig. 5C). By the protocol to synchronize cells in G1/G0 phase, 64 + 1.5% and 19 + 1.5% of wild-type MEF were in G1/G0 and S phase, respectively. Four hours after cell cycle re-entry, 20 + 1.4% and 57 + 0.8% of wild-type MEF were in G1/G0 and S phase, respectively (Fig. 5C). By striking contrast, this protocol for cell synchronization almost abolished cell cycle re-entry in STIM1^−/−^ MEF and significantly increased the cell population in sub-G1 phase. We also examined the effect of STIM1 silencing on cell proliferation by trypan blue exclusion. Our data showed that the cell growth of STIM1^−/−^MEF cells was slower than that of the wild-type MEF control, suggesting that STIM1 knockout affected cell proliferation (Fig. S13). Moreover, the immunofluorescent images showed that phosphorylation of CDK2 increased at 4 hr of cell cycle re-entry in wild-type MEFs (Fig. S14). STIM1 knockout almost abolished the phosphorylation of CDK2, regardless of S phase cell cycle re-entry. The effect of STIM1 knockdown on CDK2 phosphorylation was rescued by the subsequent transient expression of STIM1 cDNA in STIM1^−/−^ MEF (Fig. 6), suggesting that STIM1-dependent signaling is important for CDK2 activation. Taken together, these results indicate that STIM1-mediated SOCE activation directly regulated CDK2 activity during G1/S transition.

### Blockade of SOCE activation leads to cell autophagy

**Figure 7:**
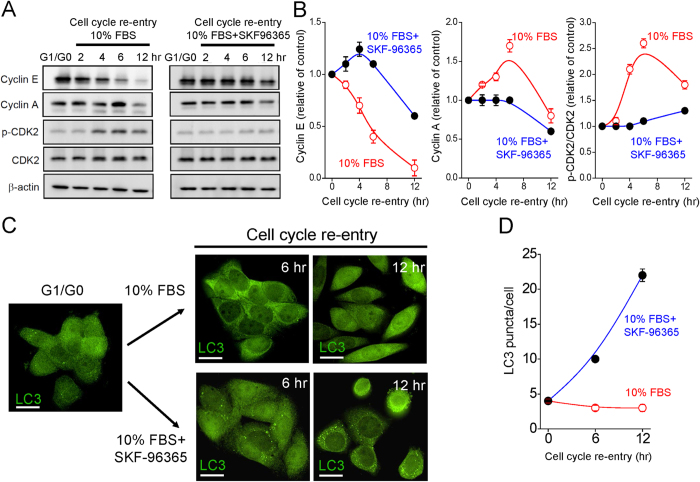
Blockade of SOCE activation leads to cell autophagy. (**A**) Western blot analysis of expression pattern of cell cycle-associated proteins, including cyclin E, cyclin A, CDK2 and phosphorylated CDK2 (p-CDK2). Cell lysates were collected from cervical cancer SiHa cells at indicated time points of cell cycle re-entry. (**B**) Densitometric quantitative analyses of cyclin E, cyclin A and p-CDK2/CDK2 level during cell cycle progression. Expression levels of cyclin E, cyclin A, CDK2 and pCDK2 were normalized against β-actin and compared with the control group (G1/G0 phase). Each point represents mean ± SEM of three different experiments. (**C**) SKF-96365 (50 μM) induces the formation of LC3 puncta at 6, 12 hrs of cell cycle re-entry. Representative images showing the expression of LC3 (green). Scale bars: 10 μm. (**D**) Quantitative analyses of cellular LC3 puncta. Each value represents mean ± SEM from at least 30 different cells.

We also analyzed the expression of the main cell cycle regulatory proteins and evaluated the activity of specific cyclin-dependent kinases (Cdk) by pharmacologic inhibition of SOCE activity (Fig. 7A,B). Compared to that of G1/G0 phase, the expression level of cyclin E declined over time when cells re-entered the cell cycle. In contrast, SKF-96365 led to the upregulated cyclin E expression up to 4 hours of S phase re-entry (Fig. 7A,B). Furthermore, cyclin A started to accumulate at the end of G1 phase, gradually increased during the S phase, attained the maximum level at 6 hours of cell cycle re-entry, and remarkably decreased at 12 hours of cell cycle re-entry (Fig. 7A,B). In contrast, the expression level of cyclin A was relatively constant by 6 hours of cell cycle re-entry when SOCE activation was inhibited by SKF-96365 (Fig. 7A,B). The expression of phosphorylated CDK2 was faint at G1/G0 phase, and increased steadily up to 6 hours of cell cycle re-entry (Fig. 7A,B). In contrast, SKF-96365 inhibited the phosphorylation of CDK2 during cell cycle re-entry (Fig. 7A,B).

Inhibition of SOCE activation during cell cycle progression induced the accumulation of cyclin E up to 4 hours of S phase re-entry (Figs 7A,B and S11), partially due to the prolonged turn-over of cyclin E in SOCE inhibited cells (Fig. S15). Whether such altering cyclin E expression leads to cell autophagy or simply proteolysis is not known. To address this question, autophagic flux during cell cycle progression in the presence or absence of SKF-96365 was analyzed. In autophagic cells, LC3-II is associated with the membrane of phagophores or autophagosomes, appearing as bright puncta in immunofluorescent staining^19^. As shown in Fig. 7C,D, SKF-96365 induced a time-dependent increase in the intensity of LC3 puncta formation, suggesting that blockade of G1/S transition induces cell autophagy.

## Discussion

This is the first study to show the fluctuating activity of SOCE during cell cycle progression and to highlight the important role of SOCE in controlling G1/S cell cycle transition. SOCE appears to be the major means of regulated Ca^2+^ influx and signal transduction in non-excitable cells^7^,^8^. As a second messenger, SOCE not only can induce a short-term cellular response, such as protein-protein interactions and granule secretion, but can also initiate longer-term regulatory mechanisms such as gene transcription that supports cell growth and apoptosis^20^,^21^,^22^,^23^. Here we reported that STIM- and Orai1-mediated SOCE regulating G1/S transition seems to be ubiquitous in many different cell types. The following evidence supports this conclusion. (1) SOCE was upregulated from G1 to S transition in different protocols to activate SOCE and in different cell lines from the epithelial or mesenchymal origin. (2) Accelerated and sustained STIM1-trafficking, real-time detected by TIRF microscopy, can partly explain the upregulated SOCE in G1/S transition. (3) The siRNA-mediated knockdown of STIM1/2 or Orai1 expression or the pharmacologic inhibition of SOCE activity significantly induced G0/G1 phase cell cycle arrest. (4) Results from MEF cells lacking STIM1 confirmed that the regulation of cell cycle G1/S transition required STIM1-dependent SOCE activation.

The importance of Ca^2+^ signaling in the regulation of cell cycle progression has been studied in different cell models, but the way in which it controls distinct checkpoints in the cell cycle yet remains elusive. Cyclin E is a nuclear protein that binds to CDK2 and forms an active complex in late G1 and leads entry into S phase^24^,^25^. Choi and colleagues demonstrated a possible calmodulin (CaM) binding site on cyclin E that could be involved in Ca^2+^-sensitive G1/S transition in vascular smooth muscle cells^26^. They reported that the kinase activity of cyclin E/CDK2 was responsive to functional changes in Ca^2+^ concentration. Nevertheless, the molecular identity that mediates Ca^2+^ signaling to control cyclin E/CDK2 activity has remained unclear. Here we show that STIM1-Orai1 pathway of SOCE plays an important role in regulating G1/S transition. STIM1 is the molecular linker from ER Ca^2+^ store depletion to the plasma membrane SOCE. STIM1 can interact with various plasma membrane Ca^2+^ channels, such as Orai proteins and the transient receptor potential channel family, to form the functional pore subunit of the SOC channel. Our results indicate that CDK2 phosphorylation is associated with STIM1-mediated SOCE activity during G1/S transition. Moreover, MEF lacking STIM1 exhibited a decreased SOCE activity at S phase and arrested in G1/S transition, compared to wild-type MEF. The phosphorylation of CDK2 as well as expression levels of SOCE-related molecules, such as STIM2 and Orai1, were also decreased in STIM1^−/−^ MEF cells. Thus, in addition to the lack of STIM1, the G1/S cell cycle transition effects of STIM1^−/−^ MEF cells might be due to the decrement of some other proteins. However, the depletion of STIM1 by siRNA showed no effect on the expression level of Orai1 or STIM2 (Fig. S12C), but successfully phenocopied the G0/G1 phase cell cycle arrest of STIM1^−/−^ MEF cells. More importantly, the STIM1 rescue study in STIM1^−/−^ MEF confirmed that STIM1-mediated SOCE activation directly mediates the activation of CDK2. These results suggest that STIM1-mediated SOCE activation is the major determinant of the Ca^2+^ sensitivity regulating G1/S transition.

In this study, we demonstrated that STIM and Orai1 underlying SOCE controls G1/S cell cycle transition through a unique mechanism that involves, at least partially, the crosstalk between autophagy and apoptosis. Autophagy is an important and conserved homeostatic mechanism that removes unnecessary proteins among eukaryotes^27^. Although autophagy and apoptosis constitute distinct cellular processes with often opposing outcomes, the role of autophagy and its crosstalk with apoptosis has been reported to be associated with cell cycle control^28^,^29^. By the dysregulation of cyclin E turnover and CDK2 phosphorylation, blockade of SOCE activity results in the cell cycle arrest at G0/G1 phase and consequently the disturbance of S phase entry. The presence of autophagosome in SOCE inhibition of cell cycle arrest could be linked to the autophagic degradation activity on the excursive expression of cyclin E. Besides, the major characteristics of apoptosis, such as cells in the subG1 phase and the presence of active caspase-3, are noted in these cells with SOCE inhibition. This implies that the failure to properly regulate cyclin E expression might cause the cell cycle arrest, which leads to cell apoptosis.

**Figure 8:**
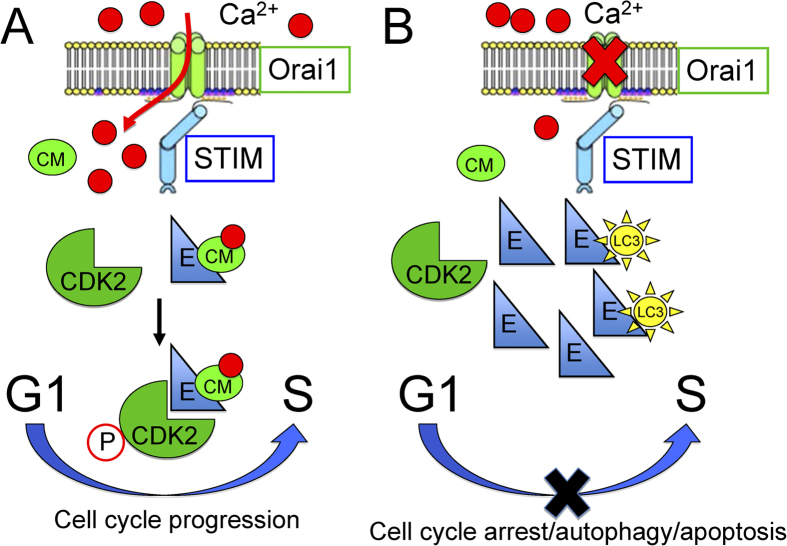
The schematic model illustrates that G1/S cell cycle transition depends on the STIM1-Orai1 pathway of SOCE. (**A**) The proposed concept on the role of SOCE–mediated Ca^2+^ signal in the regulation of G1/S cell cycle transition. The activation of SOCE controls the expression level of cyclin E and the interaction between cyclin E and phospho-CDK2 that regulate G1/S cell cycle transition. (**B**) Inhibition of SOCE activation results in the excursive expression of cyclin E that leads to cell autophagy and apoptosis. CM: calmodulin.

Taken together the results from the current and previous studies, the mechanism by which SOCE activity controlling cell cycle G1/S transition can be summarized as follows (Fig. 8). Ca^2+^ microdomain resulting from SOCE is likely the molecular basis for the Ca^2+^ sensitivity controlling cell cycle progression. The coupling between STIM1 and Orai1 is the molecular identity underlying the activation of SOCE. The activation of SOCE controls the expression levels and interaction of cell cycle associated proteins that regulate G1/S cell cycle transition (Fig. 8A). Inhibition of SOCE activation results in the excursive expression of cyclin E that leads to cell autophagy and apoptosis (Fig. 8B).

## Materials and Methods

### Cell cultures, transfection, RNA interference

Cultures of cervical cancer cell lines (SiHa, and HeLa), human osteosarcoma Epithelial cell line (U2OS), mouse embryonic fibroblasts cell line (MEF), kidney proximal tubule cell line (LLC-PK1), mouse embryonic fibroblasts (MEF) lacking STIM1 (STIM1^−/−^) and stable pools of cervical cancer cells overexpressing EGFP-STIM1 were prepared as previously described^11^,^30^,^31^. EGFP-STIM1 cDNA plasmids were transfected into SiHa cells using Lipofectamine 2000 (Invitrogen) and cells with STIM1 overexpression were selected by G418 (Sigma-Aldrich). A FACSAria cell sorter (BD Biosciences) was used to isolate the stable pools of cells overexpressing human STIM1. Two independent pairs of siRNAs (Sigma) and a siRNA pool of three different duplexes (Santa Cruz Biotechnology) targeting STIM1, STIM2, or Orai1 were used in this study.

### Cell cycle analysis

Cell-cycle stage was determined by using FACS. Cells were harvested, washed with PBS, and fixed in ice-cold 80% ethanol for 6 h, and then incubated with 1 mg/mL RNase and 20 mg/mL propidium iodide for at least 30 min. Cells were determined as in G0/G1, G2/M, and S phase based on the fluorescence intensity and the cell cycle distribution was analyzed by the Cell Fit software (BD Biosciences).

### Antibodies, chemicals, and immunoblotting

The detailed information of chemicals and antibodies are listed in Supplementary Materials and Methods. Immunoblots were detected with affinity-purified antibodies against various molecules and horseradish peroxidase-conjugated secondary antibody (Jackson ImmunoResearch). Bands in the immunoblots were quantified using ImageQuant LAS 4000 (GE Healthcare).

### Single cell [Ca^2+^]_i_ measurement

[Ca^2+^]_i_ was measured at 37 °C with the Fura-2 fluorescence ratio method on a single-cell fluorimeter, as previously described (Chiu *et al.*)^30^. Fura-2/acetoxymethyl ester (Fura-2/AM) were excited alternatively between 340 nm (*I*_*340*_) and 380 nm (*I*_*380*_) using the Polychrome IV monochromator (Till Photonics) and images were detected by the Olympus IX71 inverted microscope equipped with a xenon illumination system and an IMAGO CCD camera (Till Photonics). The fluorescence intensity of excitation at 510 nm was monitored to calculate [Ca^2+^]_i_ using the TILLvisION 4.0 program (Till Photonics).

### Immunofluorescence, confocal microscopy, and image analyses

Immunofluorescent staining were done using affinity-purified antibodies against various molecules and AlexaFluor-conjugated secondary antibodies (Invitrogen). The fluorophores were excited by laser at 405, 488, or 543 nm and detected by a scanning confocal microscope (FV-1000, Olympus). Cells were maintained in phenol red-free medium at 37 °C throughout the recording period. A pixel-by-pixel analysis by the colocalization algorithm of FV-1000 software was used to assess the colocalization of different molecules in confocal images.

### Statistical analysis

All values were reported as mean ± SEM. Student’s paired t-test or unpaired t-test was used for statistical analyses. Differences between values were considered significant when P < 0.05.

## Additional Information

**How to cite this article**: Chen, Y.-W. *et al.* The STIM1-Orai1 pathway of store-operated Ca^2+^ entry controls the checkpoint in cell cycle G1/S transition. *Sci. Rep.* **6**, 22142; doi: 10.1038/srep22142 (2016).

## Supplementary Material

Supplementary Information

Supplementary Movie 1

Supplementary Movie 2
