# Supplementary material for: The STIM1-Orai1 pathway of store-operated Ca2+ entry controls the checkpoint in cell cycle G1/S transition
Source: Sci Rep. 2016 Feb 26;6:22142. doi: 10.1038/srep22142 (PMC4768259; doi:10.1038/srep22142)
Supplement: Supplementary Information [file srep22142-s1.pdf]

The STIM1-Orai1 pathway of store-operated Ca^{2+} entry controls the checkpoint in cell cycle G1/S transition

Yun-Wen Chen[1], Yih-Fung Chen[1,5], Ying-Ting Chen[1], Wen-Tai Chiu[2], Meng-Ru Shen[1,3,4]

[1]Departments of Pharmacology, [3]Obstetrics and Gynecology, College of Medicine, [2]Department of Biomedical Engineering, [4]Advanced Optoelectronic Technology Center, College of Engineering, National Cheng Kung University, Tainan, Taiwan [5]Graduate Institute of Natural Products, College of Pharmacy, Kaohsiung University, Kaohsiung, Taiwan

Supplementary Information

Supplementary Figure S1. The fluctuation of SOCE activation is ubiquitous during cell cycle progression.

Supplementary Figure S2. The upregulation of SOCE activation is ubiquitous in G1/S cell cycle transition.

Supplementary Figure S3. Different protocols of cell cycle synchronization.

Supplementary Figure S4. SOCE is down-regulated from S to G2/M transition.

Supplementary Figure S5. No increased aneuploid was noted in ionomycin-treated group.

Supplementary Figure S6. Knockdown efficiency for the experiments done in Figure 2.

Supplementary Figure S7. Knockdown of STIM1 or STIM2 expression inhibits SOCE activation.

Supplemental Figure S8. STIM1, but not STIM2, aggregates and translocates towards cell periphery upon thapsigargin-induced ER Ca2+ depletion.

Supplementary Figure S9. Silencing Orai1 induces cell cycle arrest.

Supplementary Figure S10. Double knockdown of STIM1 and STIM2 alters cyclin E/CDK2 activity.

Supplementary Figure S11. Knockdown of Orai1 expression alters cell cycle-associated proteins.

Supplementary Figure S12. The expression levels of STIM2 and Orai1 are decreased in STIM1-/- MEF cells, but not in STIM1 knockdown cervical cancer SiHa cells.

Supplementary Figure S13. STIM1-silencing in MEF inhibits cell proliferation.

Supplementary Figure S14. Phosphorylation of CDK2 increased at 4 hrs of cell cycle re-entry in wild-type MEFs.

Supplementary Figure S15. Protein stability of cyclin E.

Supplementary movie legends
Supplementary Methods including RNA interference,Western blot analysis, Immunofluorescence, confocal microscopy, Total internal reflection fluorescence (TIRF) microscopy ,and Single cell $[Ca^{2+}]_i$ measurement

Suppl. Fig 1

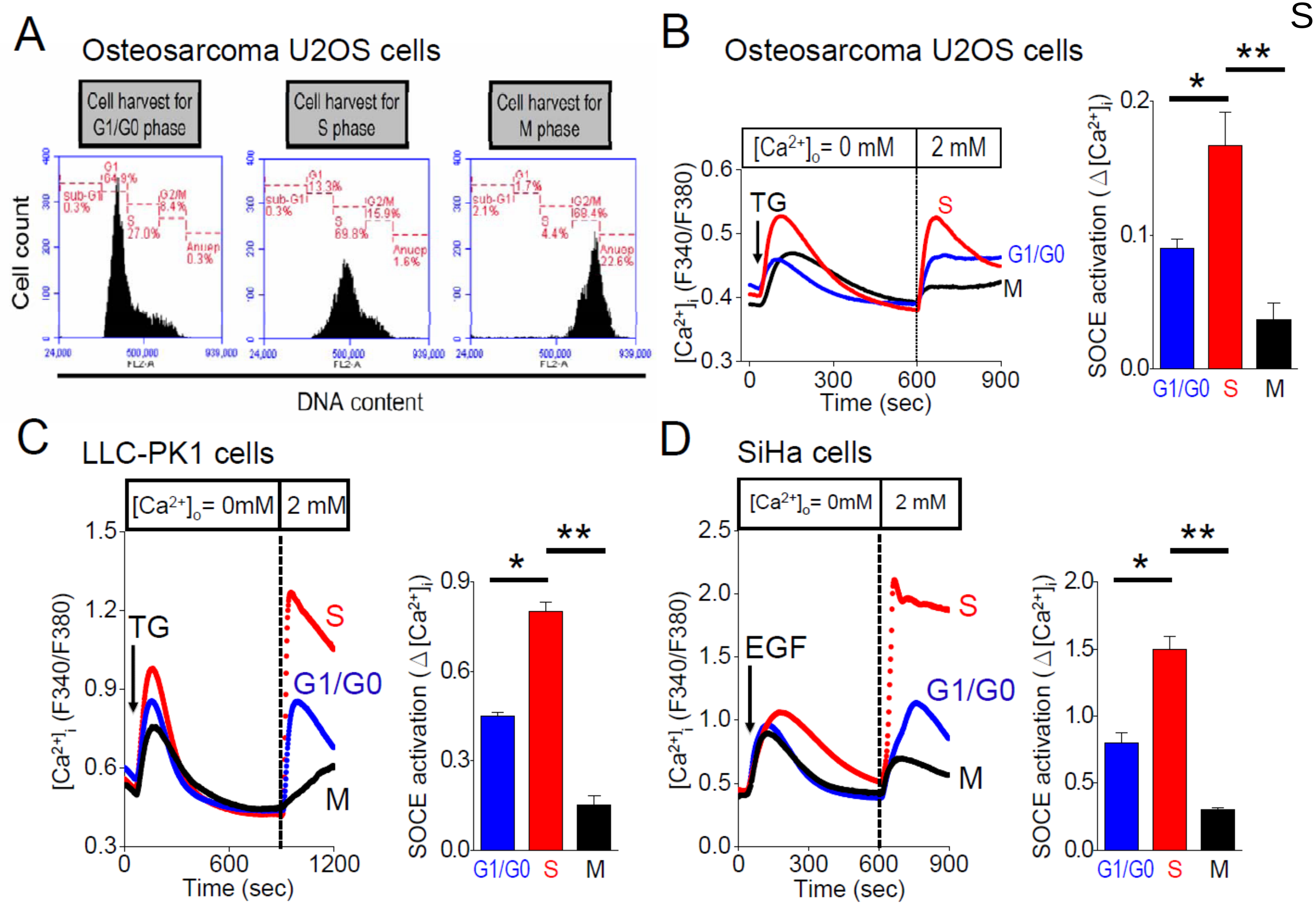

Supplementary Figure S1. The fluctuation of SOCE activation is ubiquitous during cell cycle progression. *(A)* Representative FACS measurements to determine cell cycle stages of U2OS cells. ***(B) (C)*** The SOCE activity fluctuates during cell cycle progression of osteosarcoma U2OS cells and kidney proximal tubule LLC-PK1 cells. Left panel, Representative $[Ca^{2+}]_i$ measurement. Each trace is the mean $[Ca^{2+}]_i$ measurement of at least 30 cells. Arrow, adding 2 μM thapsigargin (TG). Right panel, Quantitative analysis of SOCE at different cell cycle stage. Each value represents mean ± SEM of at least 100 cells. ***(D)*** EGF-induced activation of SOCE fluctuates during cell cycle progression of cervical cancer SiHa cells. Left panel, Representative $[Ca^{2+}]_i$ measurement. Each trace is the mean $[Ca^{2+}]_i$ measurement of at least 30 cells. Arrow, adding 100 ng/ml epidermal growth factor (EGF). Right panel, Quantitative analysis of SOCE at different cell cycle stage. Each value represents mean ± SEM of at least 100 cells. *$P < 0.01$ **$P < 0.001$ by unpaired t test.

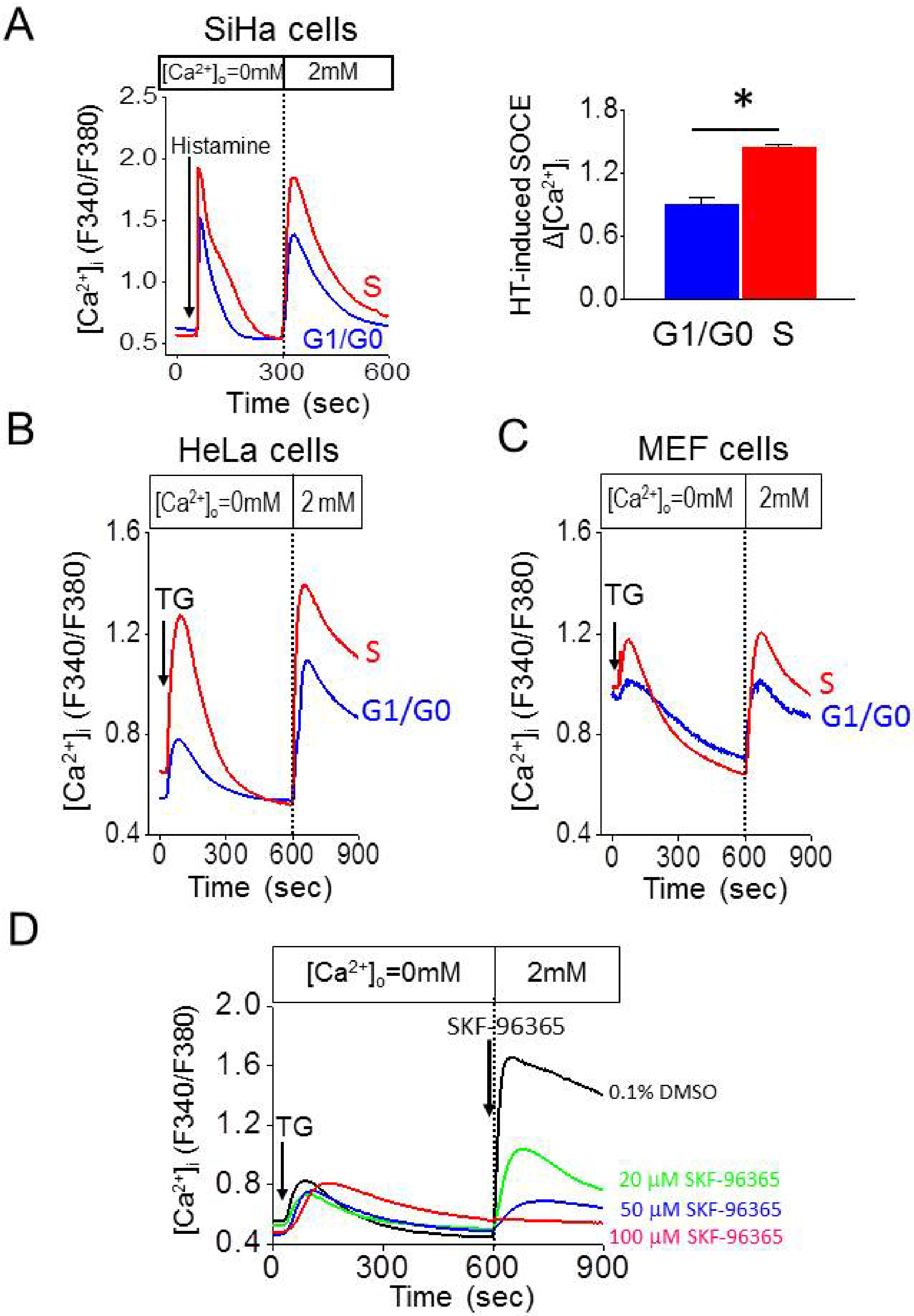

Supplementary Figure S2. The upregulation of SOCE activation is ubiquitous in G1/S cell cycle transition. ***(A)*** Different protocols to activate SOCE activation. Representative $[Ca^{2+}]_i$ measurement in cervical cancer SiHa cells. Mean traces of $[Ca^{2+}]_i$ measurement from at least 30 different cells in each experiment. Arrow, adding 100μM histamine, *P < 0.01. ***(B) (C)*** SOCE is upregulated at S phase in multiple cell lines, including cervical cancer cell line (HeLa), mouse embryonic fibroblasts (MEF). Mean traces of $[Ca^{2+}]_i$ measurement from at least 30 different cells in each experiment. Arrow, adding 2 μM thapsigargin (TG). ***(D)*** SKF-96365 does-dependently inhibits SOCE activation. Each trace was averaged from at least 30 SiHa cells.

Suppl. Fig 3

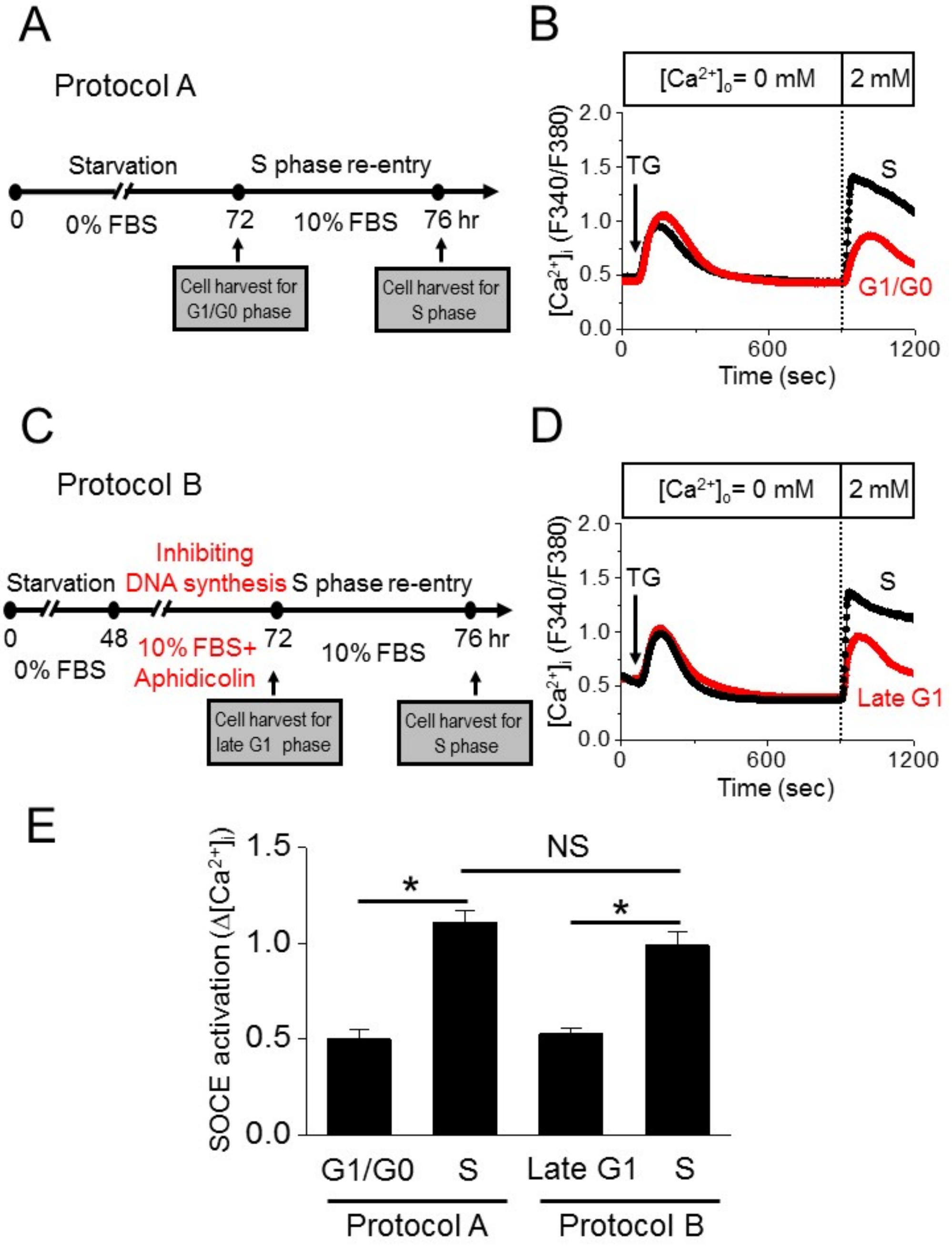

Supplementary Figure S3. Different protocols of cell cycle synchronization. ***(A)*** The protocol A of cell cycle synchronization: For G1/G0 phase: Cells grew in 0% FBS for 72 hours, then harvested for FACS measurement. For S phase: Cells grew in 0% FBS for 72 hours and then in 10% FBS for 4 hours before FACS measurement. ***(B)*** Representative $[Ca^{2+}]_i$ measurement in cervical cancer SiHa cells. Mean traces of $[Ca^{2+}]_i$ measurement from at least 30 different cells in each experiment. Arrow, adding 5 μM thapsigargin. ***(C)*** The protocol B of cell cycle synchronization: For late G1 phase: Cells grew in 0% FBS for 48 hours, then cultured in 10% FBS with 5 μM aphidicolin for 24 hours before harvest. For S phase: Cells arrested in late G1, then in 10% FBS for 4 hours before FACS measurement. ***(D)*** Representative $[Ca^{2+}]_i$ measurement in cervical cancer SiHa cells. Mean traces of $[Ca^{2+}]_i$ measurement from at least 30 different cells in each experiment. Arrow, adding 2 μM thapsigargin. ***(E)*** Quantitative analyses of SOCE activation. Each column is mean ± SEM of three independent experiments. *P<0.01, NS: non-significant.

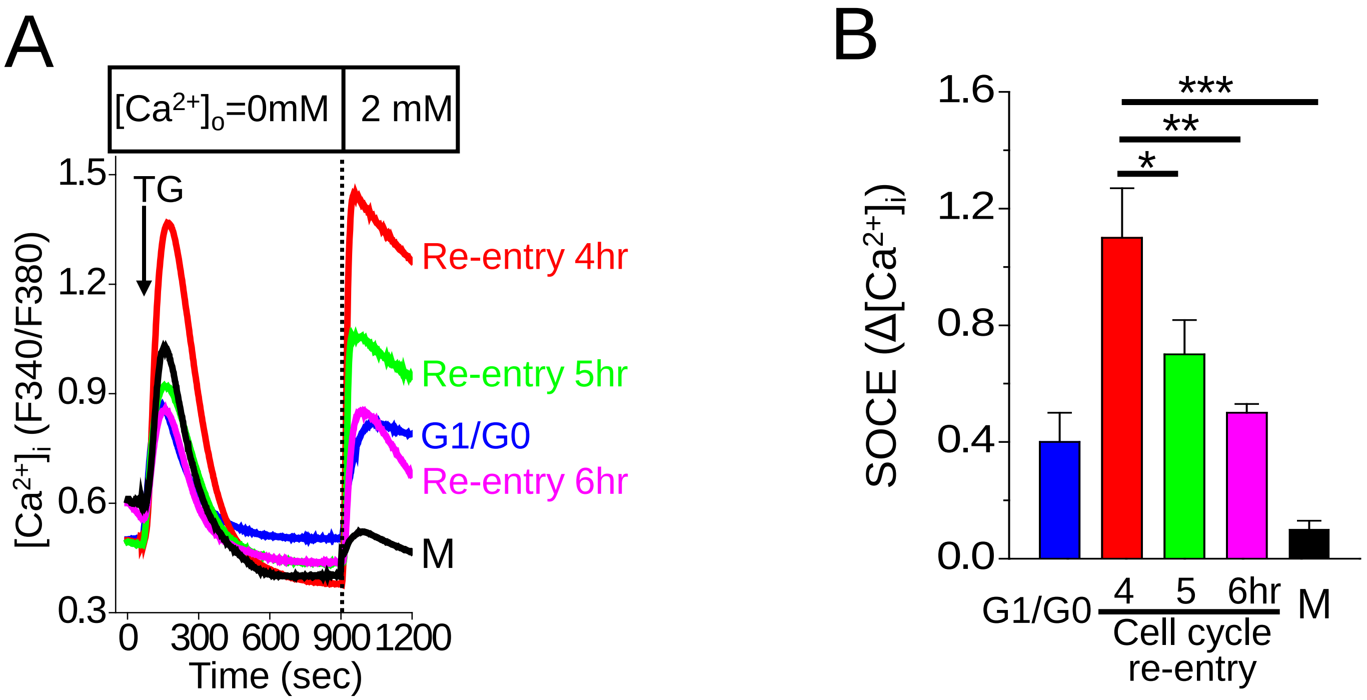

Supplementary Figure S4. SOCE is down-regulated from S to G2/M transition. ***(A)*** Representative traces of SOCE at indicated time points of cell cycle re-entry. Cervical cancer SiHa cells were first depleted of stored Ca^{2+} by 2 μM thapsigargin (TG) in the absence of external Ca^{2+} and then re-exposed to 2 mM Ca^{2+}-containing external solution, which caused a large increase in $[Ca^{2+}]_i$. ***(B)*** Quantitative analyses of SOCE activation at the indicated time point of cell cycle re-entry. Data are mean ± SEM of at least 90 cells from three independent experiments. *P<0.05, **P<0.01, ***P<0.001 by unpaired t test.

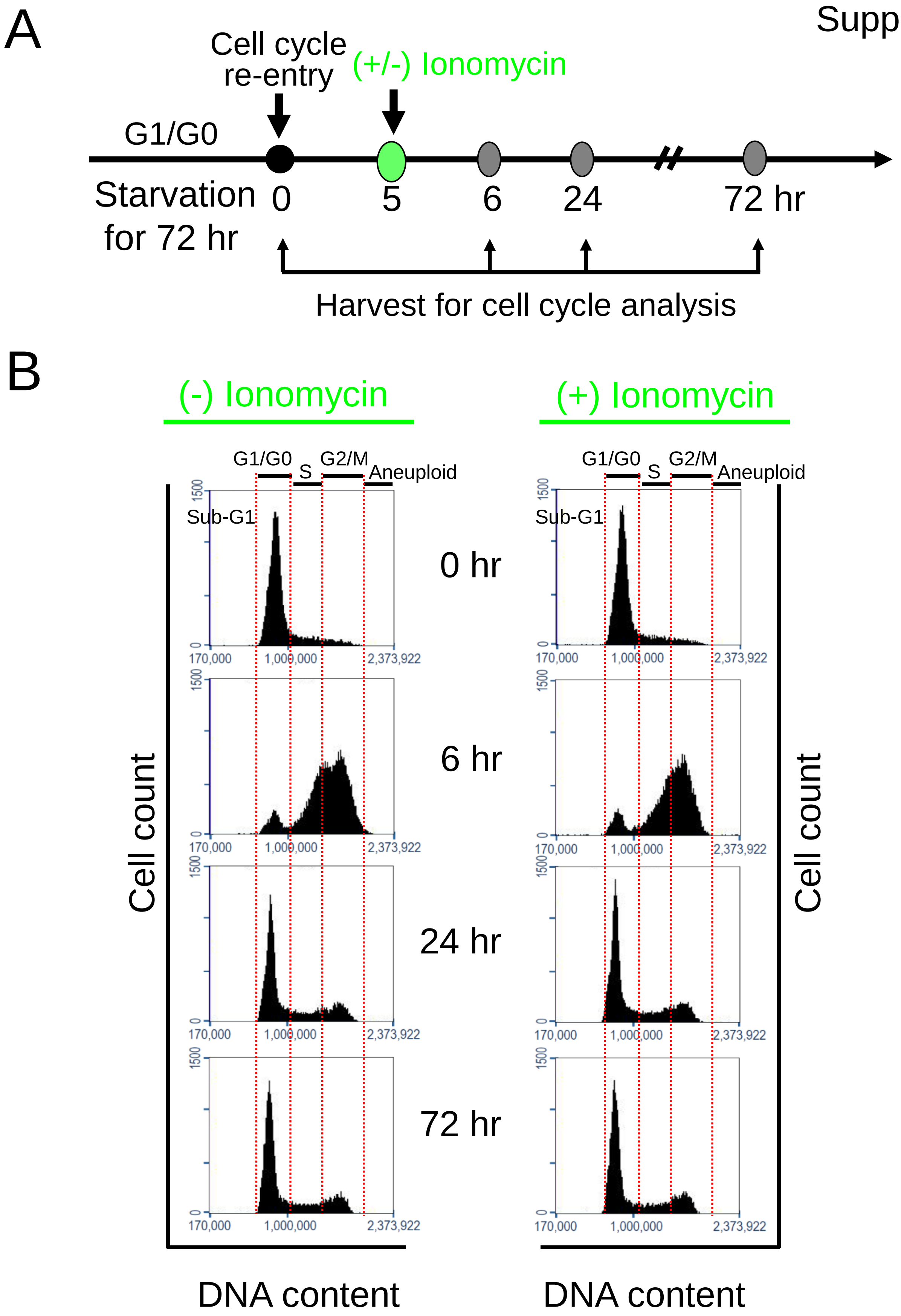

Supplementary Figure S5. No increased aneuploid was noted in ionomycin-treated group.

(A) The protocol of cell cycle synchronization to determine whether Ca^{2+} signaling is important for S to G2/M transition. Arrow, adding 0.5 μM ionomycin to maintain intracellular Ca^{2+} at 5 hrs of cell cycle reentry. ***(B)*** Representative results of FACS measurements to determine cell cycle stages of cervical cancer SiHa cells under different culture conditions.

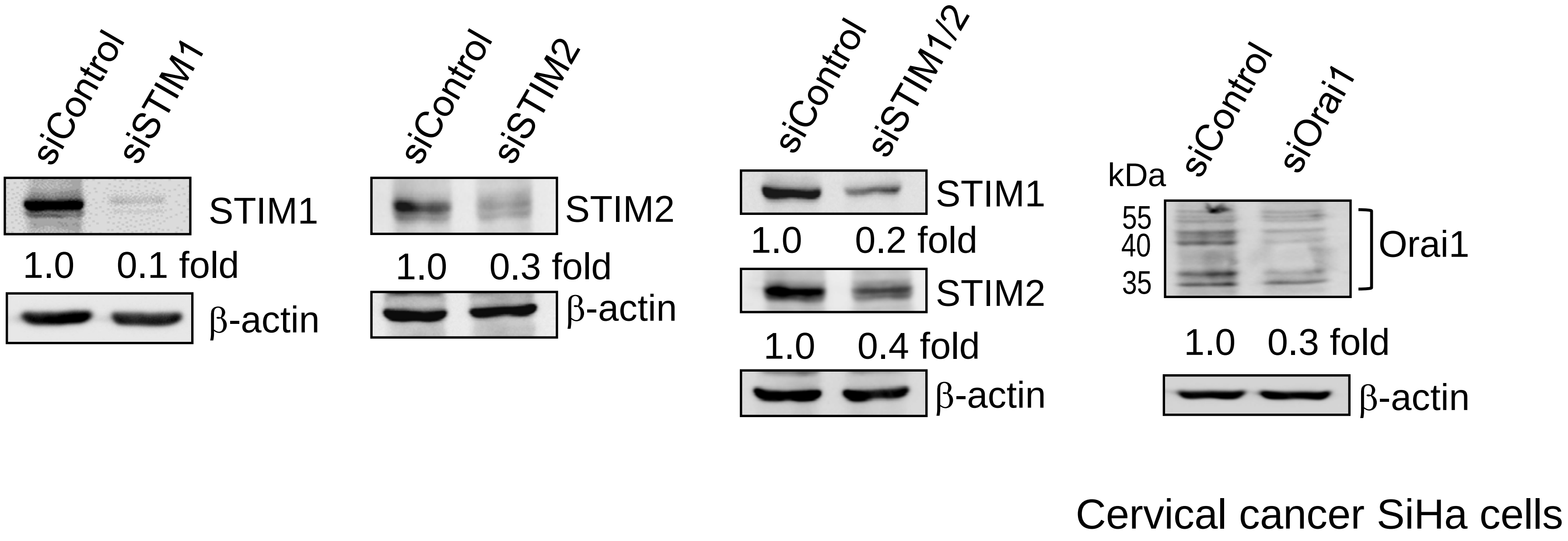

Supplementary Figure S6. Knockdown efficiency for the experiments done in Figure 2. Expression levels of STIM1, STIM2 or Orai1 were normalized by those of β-actin.

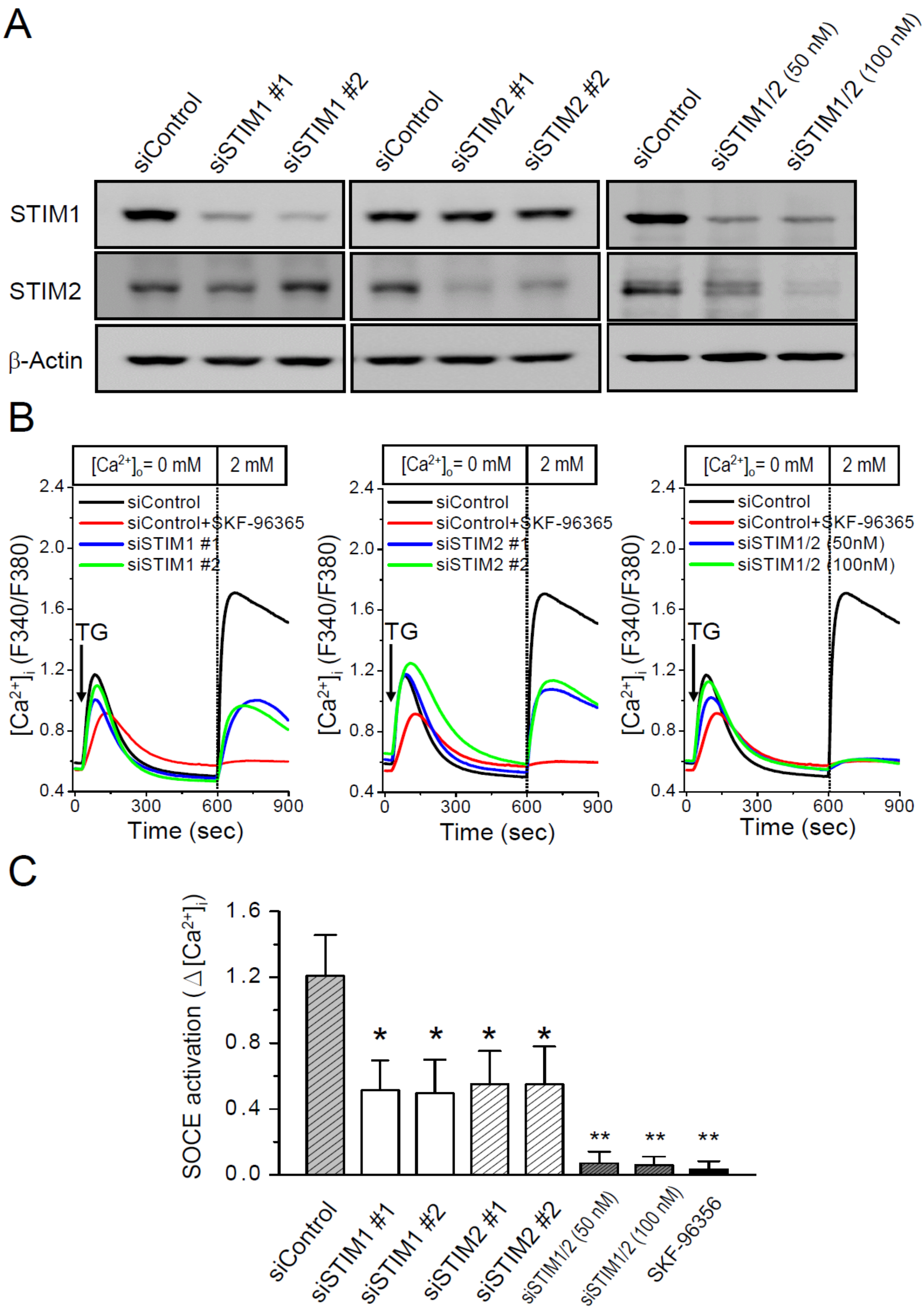

Supplementary Figure S7. Knockdown of STIM1 or STIM2 expression inhibits SOCE activation. ***(A)*** Knockdown efficiency of siControl, siSTIM1, siSTIM2, and siSTIM1/2 in cervical cancer SiHa cells. ***(B)*** The inhibitory effects of siSTIM1, siSTIM2 and 100 μMSKF96365 on SOCE activation. Mean traces of $[Ca^{2+}]_i$ measurement were from at least 60 different cells. Arrow, adding 2 μM thapsigargin. ***(C)*** Summary of SOCE activation in siControl, siSTIM1, siSTIM2, siSTIM1/2 or 100 μM SKF-96365-treated SiHa cells. Data represent mean ± SEM of at least 60 cells from three independent experiments. *P< 0.01, **P<0.001.

Suppl. Fig 8

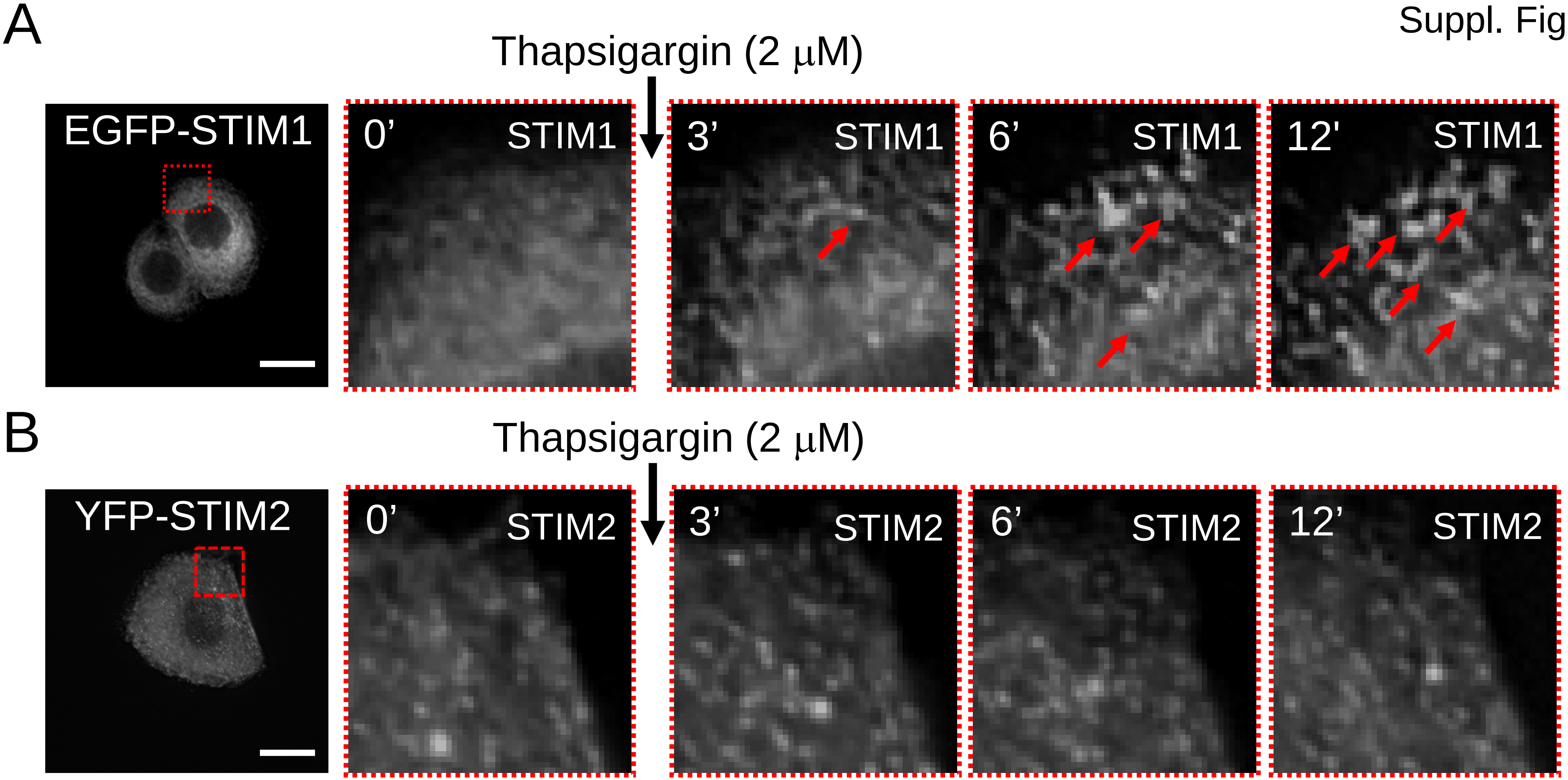

Supplemental Figure S8. STIM1, but not STIM2, aggregates and translocates towards cell periphery upon thapsigargin-induced ER Ca^{2+} depletion. Time-lapse fluorescent images of cervical cancer SiHa cells overexpressing EGFP-STIM1 (**panel A**) or YFP-STIM2 (**panel B**) were taken with the high-resolution deconvolution microscopy. Cells were stimulated by 2 μM thapsigargin at the indicated time point. Areas highlighted with red rectangles in the whole cell images were enlarged. Representative live cell images were from at least 3 different experiments. Arrow: aggregation and trafficking of STIM1. Scale bar, 15 μm.

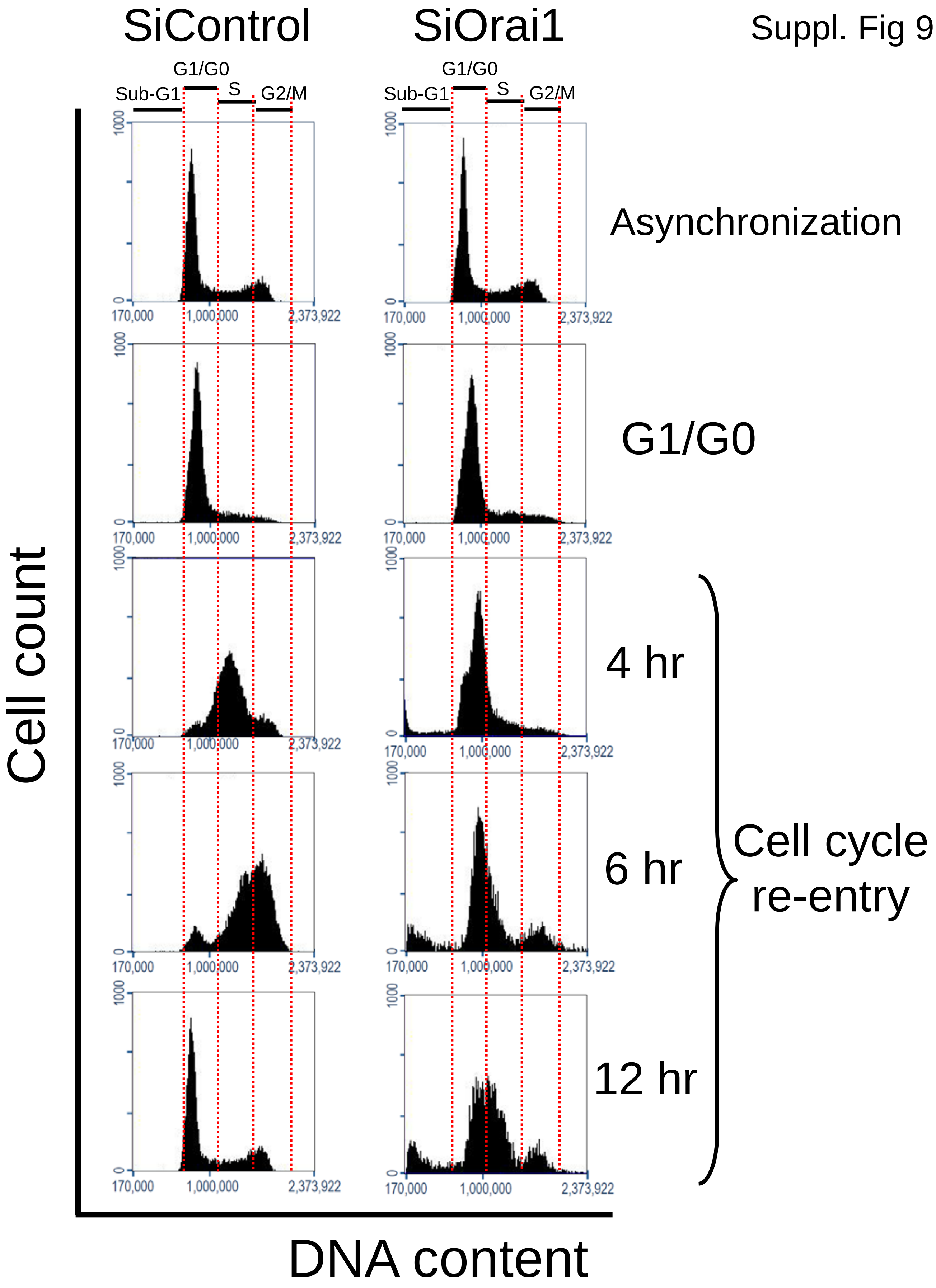

Supplementary Figure S9. Silencing Orai1 induces cell cycle arrest. Representative results of FACS measurements to determine cell cycle stages of cervical cancer SiHa cells under different culture conditions.

Suppl. Fig 10

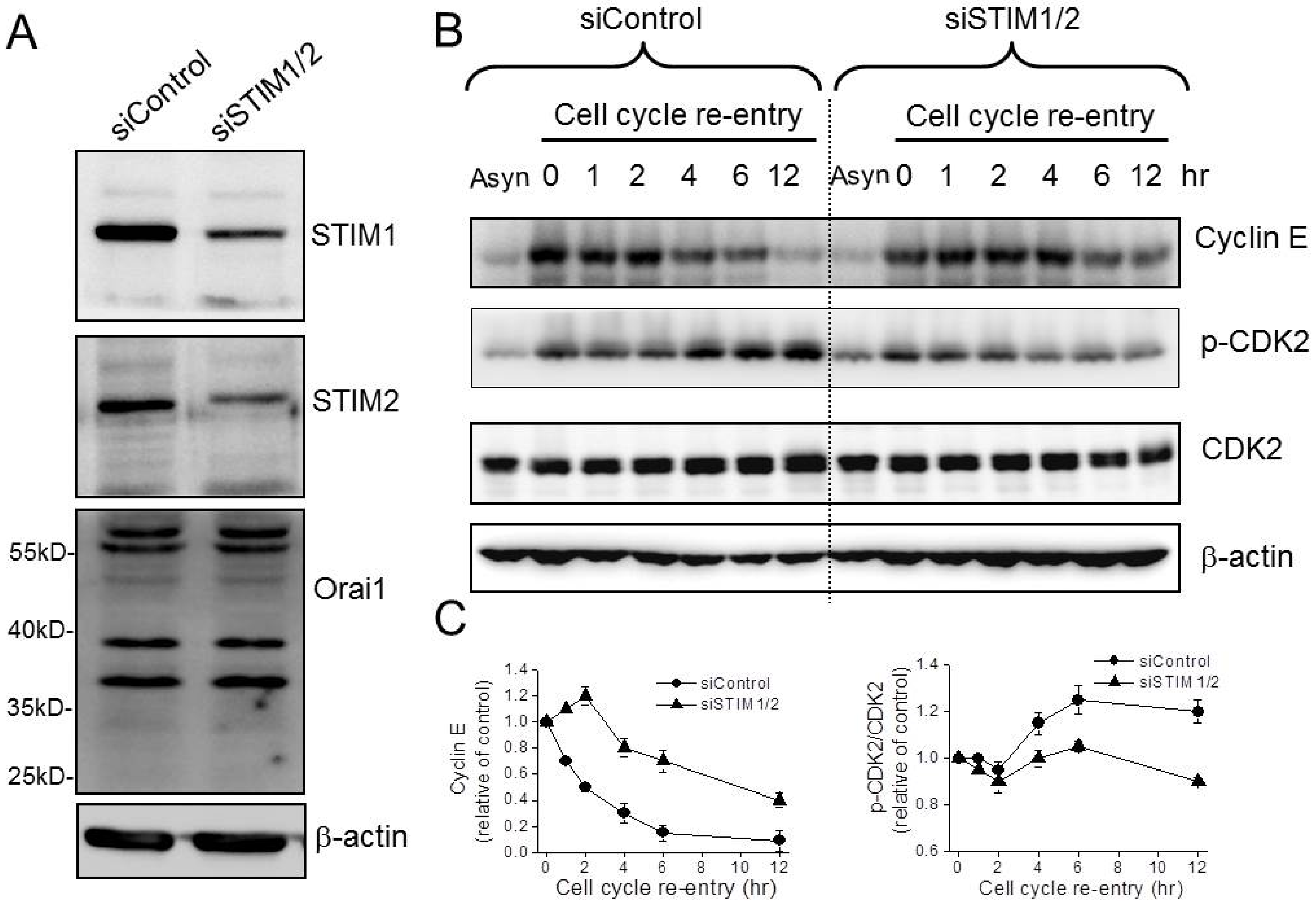

Supplementary Figure S10. Double knockdown of STIM1 and STIM2 alters cyclin E/CDK2 activity. ***(A)*** Knockdown efficiency of siControl, siSTIM1/2 in cervical cancer SiHa cells. ***(B)*** Western blot analysis of expression pattern of cell cycle-associated proteins, including cyclin E, CDK2 and phosphorylated CDK2 (p-CDK2). Cell lysates were extracted from cervical cancer SiHa cells at indicated time points of cell cycle re-entry. ***(C)*** Densitometric quantitative analyses of cyclin E, and p-CDK2/CDK2 level during cell cycle progression. Expression levels of cyclin E, CDK2 and pCDK2 were normalized against β-actin and compared with the control group (G1/G0 phase). Each point represents mean ± SEM from three different experiments.

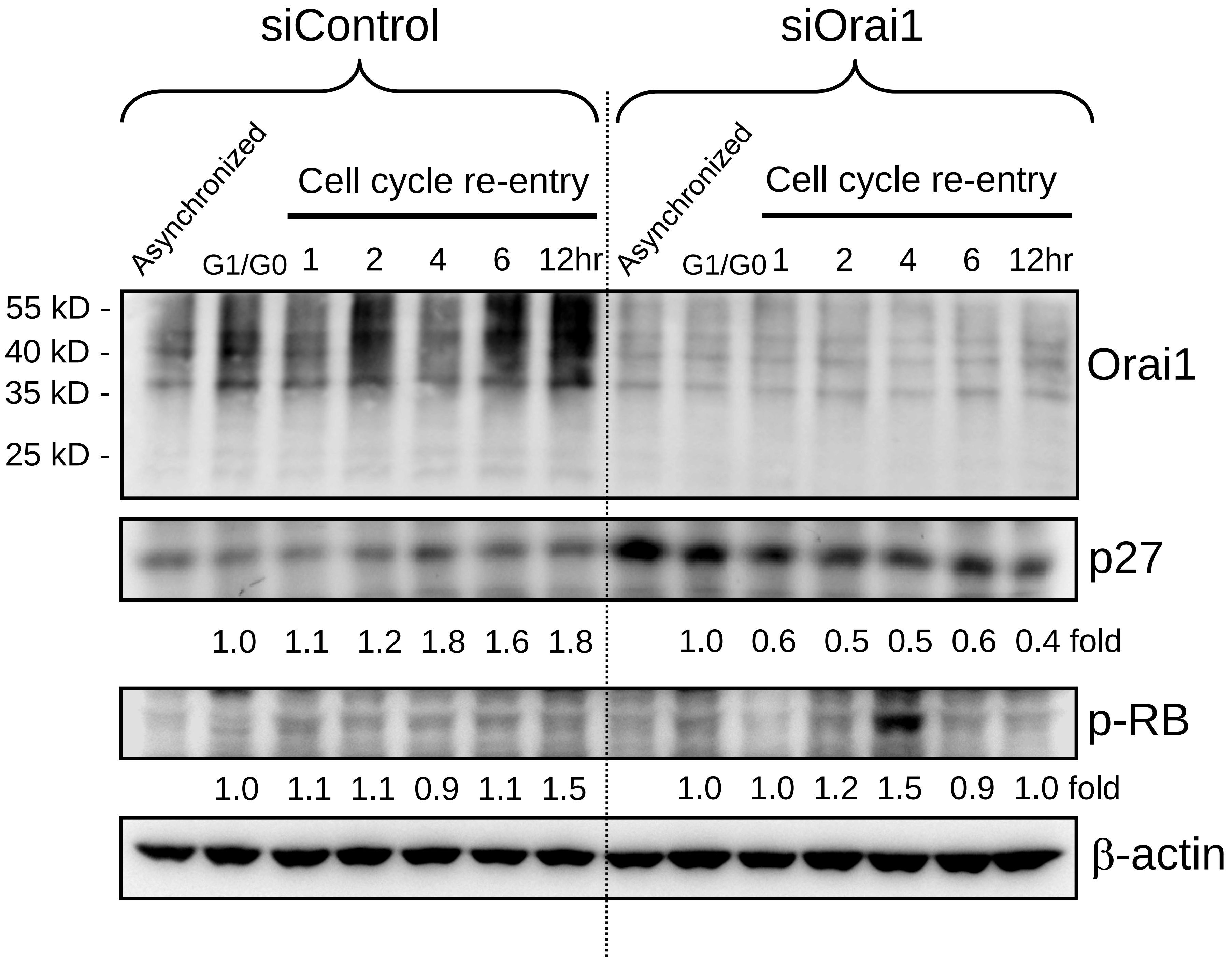

Supplementary Figure S11. Knockdown of Orai1 expression alters cell cycle-associated proteins. Western blot analysis of expression pattern of cell cycle-associated proteins, including p27 and phospho-Rb (p-RB). Cell lysates were extracted from cervical cancer SiHa cells at indicated time points of cell cycle re-entry. Expression levels of p27 and p-Rb were normalized against β-actin and compared with the control group (G1/G0 phase). The representative immunoblots were from three different experiments.

Suppl. Fig 12

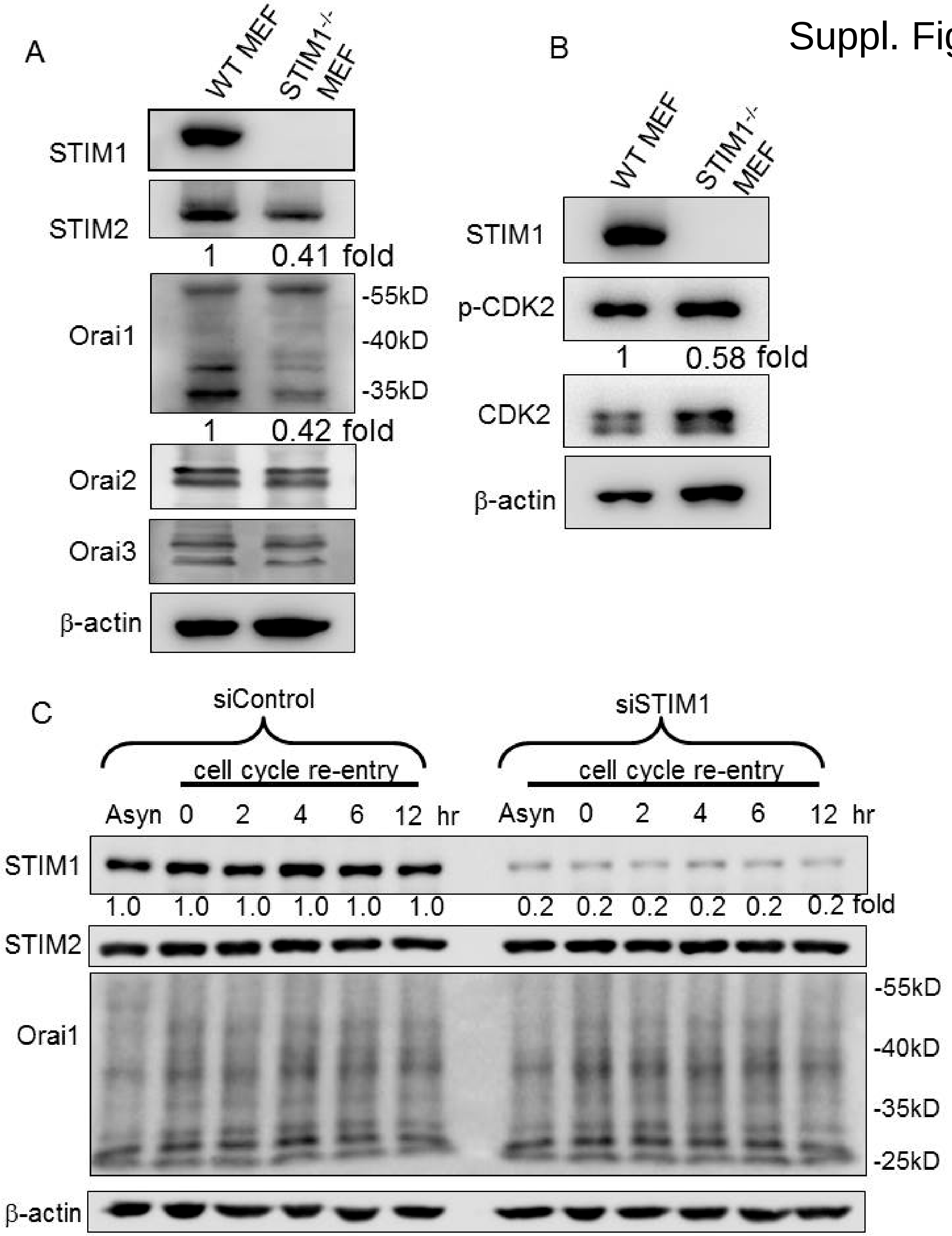

Supplementary Figure S12. The expression levels of STIM2 and Orai1 are decreased in STIM1$^{-/-}$ MEF cells, but not in STIM1 knockdown cervical cancer SiHa cells. ***(A)*** Western blot analysis of expression pattern of STIM1, STIM2, Orai1, Orai2. and Orai3 in wild-type mouse embryonic fibroblasts (MEF) and MEF lacking STIM1 (STIM1$^{-/-}$ MEF).. ***(B)*** Western blot analysis of expression pattern of CDK2 and phosphorylated CDK2 (p-CDK2) Orai3 in wild-type mouse embryonic fibroblasts (MEF) and MEF lacking STIM1 (STIM1$^{-/-}$ MEF). ***(C)*** Western blot analysis of expression pattern of STIM1, STIM2, Orai1 in siControl and siSTIM1 knockdown cervical cancer SiHa cells. A representative immunoblots were from three different experiments.

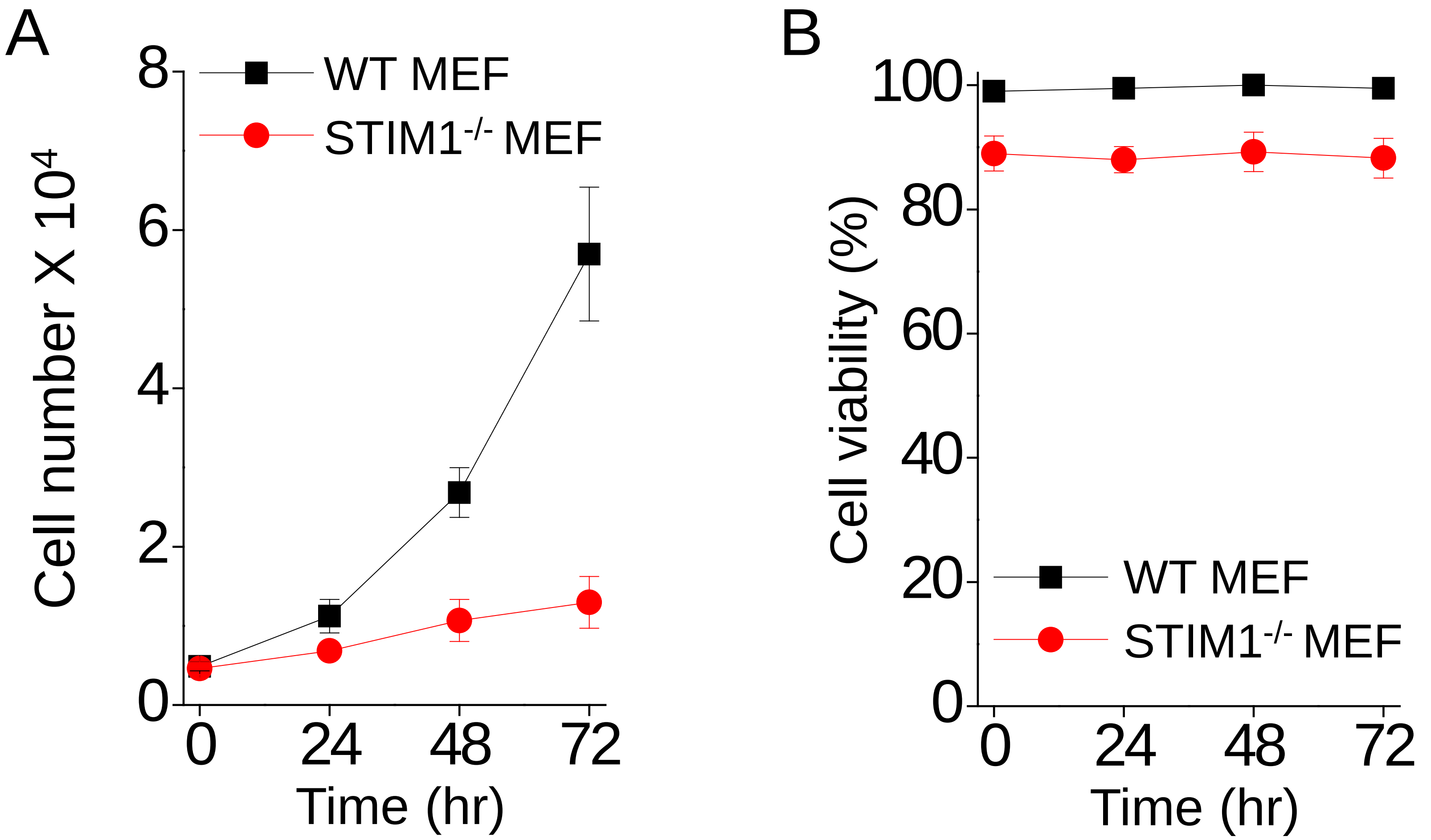

Supplementary Figure S13. STIM1-silencing in MEF inhibits cell proliferation. ***(A)*** Cell proliferation of wild-type and STIM$^{-/-}$ MEF. The cells were harvested at the indicated time, and living cells not stained with trypan blue were counted. Data represent the averages of three independent experiments. ***(B)*** Cell viability was determined by trypan blue exclusion. Data represent the averages of three independent experiments.

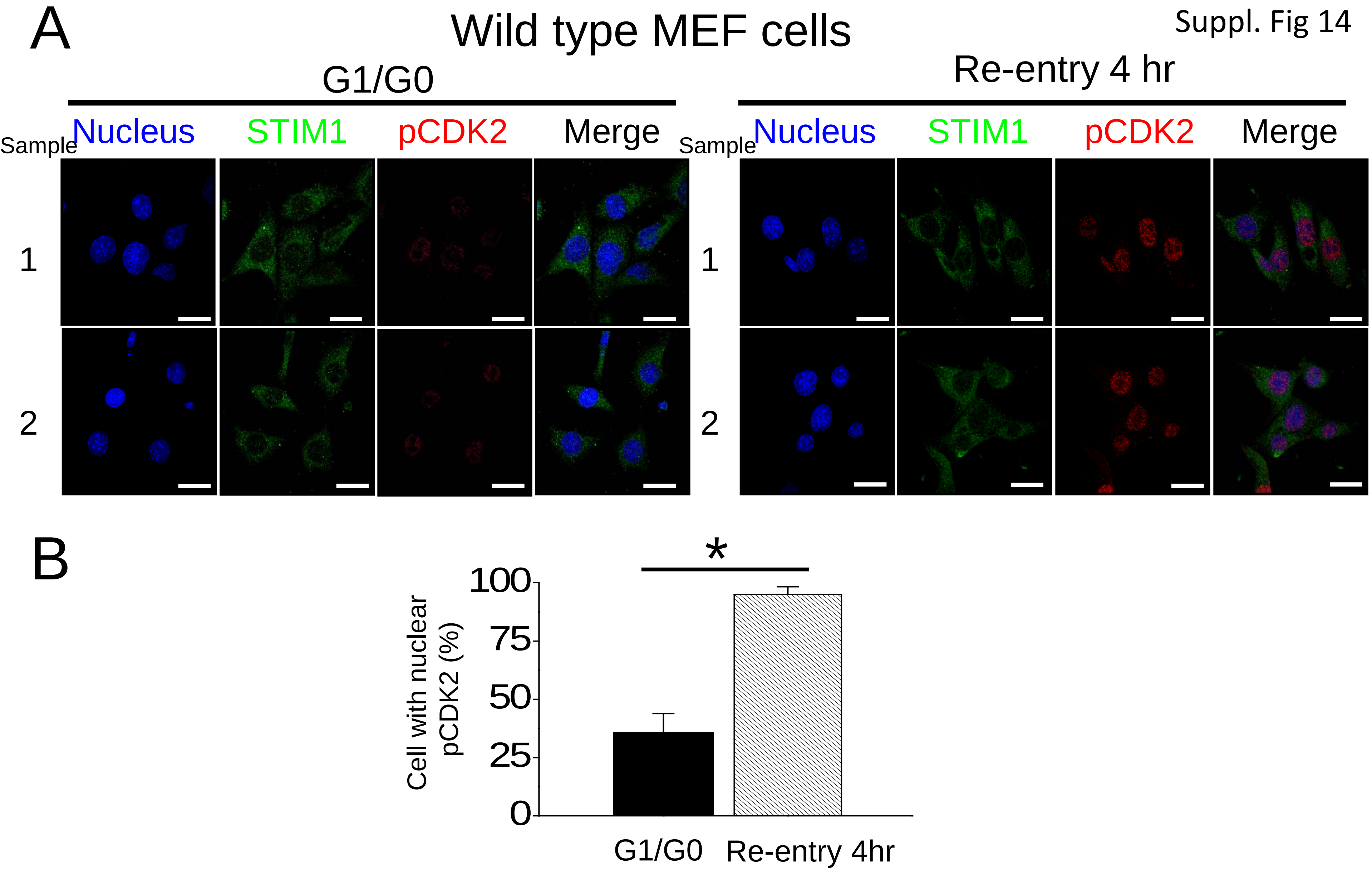

Supplementary Figure S14. Phosphorylation of CDK2 increased at 4 hrs of cell cycle re-entry in wild-type MEFs. *(A)* Representative confocal images showing the expression of phosphorylated CDK2 (pCDK2) in wild-type MEFs. Images were analyzed at G1/G0 and 4 hrs of cell cycle re-entry. Nuclei, Hoechst 33258 (blue), STIM1 (green), pCDK2 (red). ***(B)*** Quantitative analyses of pCDK2 fluorescent intensity at nuclear region at G1/G0 and 4 hrs of cell cycle re-entry in wild-type MEFs. Each value represents mean ±SEM from at least 30 different cells. *P < 0.01.

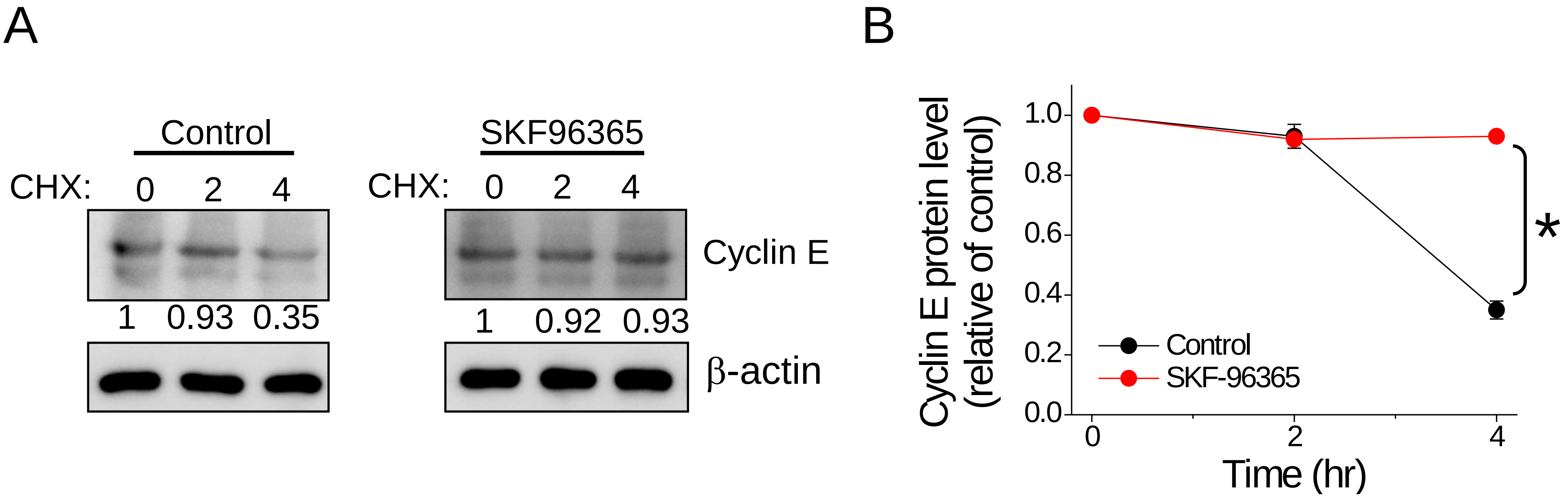

Supplementary Figure S15. Protein stability of cyclin E. ***(A)*** Cervical cancer SiHa cells were preincubated with the vehicle control or 50μM SKF-96365 before the treatment of the translational inhibitor cycloheximide (CHX, 20 μg/mL). The representative immunoblots were from three different experiments. ***(B)*** Densitometric quantification of cyclin E protein levels. Points, mean ± SEM; *P < 0.01, compared with control group.

Supplementary Movie legends

Supplementary Movie 1. STIM1 trafficking at G1/G0 phase. Time-lapse TIRF image recording of cervical cancer SiHa cells stably expressing EGFP-STIM1 upon thapsigargin stimulation at G1/G0 phase. SiHa cells were imaged, with exposure time ranging from 300-500 ms and 5 s interval between frames for 10 min in TIRF microscopy. Thapsigargin was added at 2 mins to the final concentration of 2 μM. Scale bar, 10 μm.

Supplementary Movie 2. STIM1 trafficking at S phase. Time-lapse TIRF image recording of cervical cancer SiHa cells stably expressing EGFP-STIM1 upon thapsigargin stimulation at S phase. SiHa cells were imaged, with exposure time ranging from 300-500 ms and 5 s interval between frames, for 10 min in TIRF microscopy. Thapsigargin was added at 2 mins to the final concentration of 2 μM. Scale bar, 10 μm.

Supplementary Methods

Primary antibodies and reagents.

Antibodies against STIM1 were from BD Transduction Laboratories. Antibodies against STIM2, CDK2, p-CDK2 and pRb were from Cell Signaling Technology. Antibodies against β-actin (clone AC-15) was from Sigma-Aldrich. Antibodies against Orai1 were from ProSci Inc. Antibodies against Orai2, Orai3, p27, cyclin A, cyclin E and LC3 were from Santa Cruz Biotechnology, Inc. Histamine, EGF, and cycloheximide were from Sigma-Aldrich. SKF96365 and lonomycin were from Cayman Chemical. mag-Fura-2/AM, Fura-2/AM and Ca^{2+}-free Dulbecco's modified Eagle's medium (DMEM) was purchased from Invitrogen

RNA interference.

For siRNA knockdown of STIM1, STIM2, Orai1, Orai2, and Orai3, cervical cancer cells were transfected with 50, and 100 nM of either targeting siRNA or a Stealth RNAi Negative Control siRNA (Invitrogen) using Lipofectamine 2000 (Invitrogen) for 72 or 96 h. Preliminary results indicated that a siRNA concentration of 100 nM for 48 h exerted the maximum inhibition of mRNA and protein expression for STIM1, STIM2, Orai1, Orai2, or Orai3 without affecting the cell viability. Two independent pairs of different siRNAs (Sigma) and a siRNA pool of three duplexes (Santa Cruz Biotechnology) targeting STIM1, STIM2, or Orai1 were used in this study.

Western blotting.

Cells were harvested with ice-cold modified RIPA buffer containing a protease inhibitor mixture (Roche Diagnostics), 100 mmol/L KCl, 80 mmol/L NaF, 10 mmol/L EGTA, 50 mmol/L h-glycerophosphate, 10 mmol/L p-nitrophenyl phosphate, 1 mmol/L vanadate, 0.5% sodium deoxycholate, and 1% Nonidet P-40. Protein concentrations were determined with the use of a Bio-Rad protein assay. Small aliquots (20-50μg) of homogenized cell sample were subjected to 7.5% SDS/PAGE, and then transferred to nitrocellulose membranes (Pall). Immunoblots were blocked, incubated with the primary antibody, washed, and incubated with the corresponding horseradish peroxidase-conjugated secondary antibody (Jackson ImmunoResearch). Bands in the immunoblots were quantified using Vision WorksLS software (UVP).

Immunofluorescence, confocal microscopy, and image analyses.

For immunofluorescent staining, cells were seeded on glass coverslips. Cells were fixed with 4% paraformaldehyde, permeabilized with 0.1% Triton X-100 for 15 min, and blocked with commercial blocking serum (Invitrogen). The cells were incubated with primary antibody at 4°C overnight, washed, and then incubated with Alexa-Fluor-conjugated secondary antibodies (Invitrogen) for 1 hour at room temperature. To detect nuclei, cells were stained with Hoechst 33258 (Invitrogen) for 1 hour at room temperature. The cells were then washed and mounted, and the fluorophores were excited by a laser at 405, 488, or 543 nm and examined with a scanning confocal microscope (FV-1000, Olympus). For the live cell imaging, cellswith EGFP-STIM1 overexpression were directlyactivated by laser at 488nm for the detection ofEGFP, respectively. Cells were maintained inphenol red-free medium at 37 °C throughout the recording period. The fluorescent Ca^{2+} indicator Fluo2/AM (Invitrogen) was used to estimate cytosolic Ca^{2+} levels. The colocalization of different molecules in confocal images was assessed using pixelby-pixel analysis and FV-1000 software.

Total internal reflection fluorescence (TIRF) microscopy.

Cervical cancer SiHa cells overexpressing EGFP-STIM1 were plated onto 35 mm glass-bottom dish (MatTek, catalog number P35G-1.5-20-C) overnight. Living cell images were acquired using an Olympus Xcellence imaging system comprising IX81 microscope, 60X 1.49 NA apochromat TIRF objective, MT-20 illumination unit, 488 nm/20 mW and 561 nm/25 mW lasers and Ando DU897E EMCCD. Image was collected at 2-second interval for 15 minutes. Avizo 3D imaging and analysis software (Mercury computer systems) were used for image analyses.

Single cell $[Ca^{2+}]_i$ measurement

Intracellular Ca^{2+} concentration ($[Ca^{2+}]_i$) was measured at 37°C with the Fura-2 fluorescence ratio method on a single-cell fluorimeter as previously described. Briefly, cells attached on glass-bottom dishes were loaded with 2 μM Fura-2/acetoxymethyl ester (Fura-2/AM) in serum-free culture medium at 37 °C for 30 min. Cells were then washed three times with PBS. The dish was then placed on the stage of an Olympus IX71 inverted microscope equipped with a xenon illumination system and an IMAGO CCD camera (Till Photonics). The Fura-2 was excited alternatively between 340 nm (I_{340}) and 380 nm (I_{380}) using the Polychrome IV monochromator (Till Photonics) and images were detected by the Olympus IX71 inverted microscope equipped with a xenon illumination system and an IMAGO CCD camera (Till Photonics). Thefluorescence intensity of excitation at 510 nm was monitored to calculate $[Ca^{2+}]i$ by TILLvisION 4.0 program (Till Photonics).IntracellularCa^{2+} concentration was calculated as previously described (Grynkiewicz et al., 1985)

Reference

Grynkiewicz G, Poenie M, Tsien RY (1985) A new generation of Ca^{2+}indicators withgreatly improved fluorescence properties. J Biol Chem 260:3440e3450.
